# Low-density granulocytes are related to shorter pregnancy duration but not to interferon alpha protein blood levels in systemic lupus erythematosus

**DOI:** 10.1186/s13075-023-03092-w

**Published:** 2023-06-22

**Authors:** Agnes Torell, Marit Stockfelt, Gunilla Larsson, Kaj Blennow, Henrik Zetterberg, Dag Leonard, Lars Rönnblom, Muna Saleh, Christopher Sjöwall, Helena Strevens, Andreas Jönsen, Anders A. Bengtsson, Estelle Trysberg, Maria Majcuk Sennström, Agneta Zickert, Elisabet Svenungsson, Iva Gunnarsson, Karin Christenson, Johan Bylund, Bo Jacobsson, Anna Rudin, Anna-Carin Lundell

**Affiliations:** 1 Department of Rheumatology and Inflammation Research, Institute of Medicine, Sahlgrenska Academy at the University of Gothenburg, Guldhedsgatan 10A, Gothenburg, 405 30 Sweden; 2 Rheumatology, Sahlgrenska University Hospital, Gothenburg, Sweden; 3 Department of Psychiatry and Neurochemistry, Institute of Neuroscience and Physiology, Sahlgrenska Academy at the University of Gothenburg, Mölndal, Sweden; 4 Clinical Neurochemistry Laboratory, Sahlgrenska University Hospital, Mölndal, Sweden; 5 Department of Neurodegenerative Disease, UCL Institute of Neurology, Queen Square, London, UK; 6 UK Dementia Research Institute at UCL, London, UK; 7 Hong Kong Center for Neurodegenerative Diseases, Clear Water Bay, Hong Kong, China; 8 Winsconsin Alzheimer’s Disease Research Center, University of Wisconsin School of Medicine and Public Health, University of Wisconsin-Madison, Madison, WI USA; 9 Department of Medical Sciences, Rheumatology, Uppsala University, Uppsala, Sweden; 10 Division of Inflammation and Infection, Department of Biomedical and Clinical Sciences, Linköping University, Linköping, Sweden; 11 Department of Obstetrics and Gynecology, Institute of Clinical Sciences, Skåne University Hospital, Lund, Sweden; 12 Department of Clinical Sciences Lund, Rheumatology, Lund University, Skåne University Hospital, Lund, Sweden; 13 Department of Womens and Childrens Health, Division for Obstetrics and Gynecology, Karolinska University Hospital, Karolinska Institute, Stockholm, Sweden; 14 Department of Medicine Solna, Division of Rheumatology, Karolinska Institute, Karolinska University Hospital, Stockholm, Sweden; 15 Department of Oral Microbiology and Immunology, Institute of Odontology, Sahlgrenska Academy at the University of Gothenburg, Gothenburg, Sweden; 16 Department of Obstetrics and Gynecology, Institute of Clinical Sciences, Sahlgrenska Academy at the University of Gothenburg, Gothenburg, Sweden; 17 Department of Obstetrics and Gynecology, Sahlgrenska University Hospital, Gothenburg, Sweden; 18 Department of Genetics and Bioinformatics, Domain of Health Data and Digitalisation, Institute of Public Health, Oslo, Norway

**Keywords:** Systemic lupus erythematosus (SLE), Neutrophils, Pregnancy, Interferon alpha, Autoantibody(ies)

## Abstract

**Background:**

An increased risk of pregnancy complications is seen in women with systemic lupus erythematosus (SLE), but the specific immunopathological drivers are still unclear. Hallmarks of SLE are granulocyte activation, type I interferon (IFN) overproduction, and autoantibodies. Here we examined whether low-density granulocytes (LDG) and granulocyte activation increase during pregnancy, and related the results to IFNα protein levels, autoantibody profile, and gestational age at birth.

**Methods:**

Repeated blood samples were collected during pregnancy in trimesters one, two, and three from 69 women with SLE and 27 healthy pregnant women (HC). Nineteen of the SLE women were also sampled late postpartum. LDG proportions and granulocyte activation (CD62L shedding) were measured by flow cytometry. Plasma IFNα protein concentrations were quantified by single molecule array (Simoa) immune assay. Clinical data were obtained from medical records.

**Results:**

Women with SLE had higher LDG proportions and increased IFNα protein levels compared to HC throughout pregnancy, but neither LDG fractions nor IFNα levels differed during pregnancy compared to postpartum in SLE. Granulocyte activation status was higher in SLE relative to HC pregnancies, and it was increased during pregnancy compared to after pregnancy in SLE. Higher LDG proportions in SLE were associated with antiphospholipid positivity but not to IFNα protein levels. Finally, higher LDG proportions in trimester three correlated independently with lower gestational age at birth in SLE.

**Conclusion:**

Our results suggest that SLE pregnancy results in increased peripheral granulocyte priming, and that higher LDG proportions late in pregnancy are related to shorter pregnancy duration but not to IFNα blood levels in SLE.

**Supplementary Information:**

The online version contains supplementary material available at 10.1186/s13075-023-03092-w.

## Background

Systemic lupus erythematosus (SLE) is a chronic inflammatory disease that affects women nine times more frequently than men, and disease onset is common in fertile ages [1]. Women with SLE are at increased risk of adverse pregnancy outcomes, including preterm birth, low birth weight, and preeclampsia compared to the general population [2–5]. They also face a higher risk of disease flares during pregnancy [6]. Risk factors for adverse outcomes in SLE pregnancy include maternal flares, presence of antiphospholipid antibodies (aPL), thrombocytopenia and low levels of the complement proteins C3 and C4 during pregnancy, and a history of lupus nephritis [4, 7–10]. Still, specific immunopathological mechanisms that could precede pregnancy complications in SLE remain to be identified.

Neutrophils are key effector cells in acute inflammation and are involved in SLE pathogenesis [11]. Activated neutrophils possess powerful effector functions including neutrophil extracellular trap (NET) formation that can be harmful to endothelial cells [12], and neutrophil activation is associated with vascular inflammation and dysfunction in SLE [13]. In uncomplicated SLE pregnancies, a longitudinal transcriptomic profiling of blood showed upregulation of gene signatures related to neutrophil pathways compared to non-pregnant women with SLE [14]. SLE pregnancies complicated by preeclampsia displayed the earliest up-regulation of the neutrophil gene signature, which was related to increased immature neutrophils in blood [14]. Low-density neutrophils or granulocytes (LDG) were first described in SLE, and LDG frequencies relate to disease activity [15, 16]. In SLE, LDG are thought to be more proinflammatory and to spontaneously form NETs compared to normal-density granulocytes (NDG) [17], but there is a debate about the origin of LDG, and whether they represent immature cells, mature and activated cells, or a mix [18]. We recently reported that women with SLE have higher proportions and more activated LDG in their blood compared to healthy women at delivery [19]. Yet, it remains to be examined if LDG proportions and granulocyte activation are increased throughout SLE pregnancies and if these factors differ during compared to after pregnancy in SLE.

Activation of the type I interferon (IFN) system is a common feature in SLE as demonstrated by increased expression of IFN-regulated genes in blood and tissues, an IFN signature, and by elevated IFNα protein concentrations in blood compared to healthy controls [20–22]. With the use of an ultrasensitive single molecule array (Simoa) digital enzyme-linked immunosorbent assay (ELISA), it has been shown that IFNα blood levels relate to disease activity and risk of relapse in SLE [23, 24]. However, IFNα protein has previously not been measured in SLE pregnancies, thus it is not known if IFNα concentrations are influenced by pregnancy.

Even if the cellular sources of excessive IFNα production in SLE are debated [25], there are several feasible mechanisms for type I IFN production. Immune complexes, consisting of autoantibodies and nucleic acid binding proteins, may trigger plasmacytoid DCs (pDCs) to secrete IFNα in a TLR7- and 9-dependent manner [26, 27]. Similarly, NETs contain endogenous nucleic acids that can trigger pDCs to secrete IFNα [28]. Indeed, antibody positivity for RNA binding proteins, but not phospholipids, is related to a high IFN signature and elevated IFNα protein levels in non-pregnant patients with SLE [24, 29]. Increased LDG proportions, on the other hand, are associated both with a high IFN signature and with aPL positivity in non-pregnant patients with SLE [16, 30, 31]. The relationship between LDG, IFNα protein levels, and aPL has not previously been investigated in SLE pregnancies.

The aim of this study was to investigate whether women with SLE have higher LDG proportions and increased granulocyte activation in blood during pregnancy compared to after pregnancy, i.e., in the late postpartum period, and if these granulocyte-related variables are associated with IFNα protein levels, autoantibody profile and/or pregnancy duration in SLE.

## Patients and methods

### Study cohort

In this longitudinal multicenter study, pregnant women with SLE (*n* = 69) were recruited at five Rheumatology clinics in Sweden: Gothenburg (Sahlgrenska University Hospital, *n* = 20), Stockholm (Karolinska University Hospital, *n* = 35), Uppsala (Uppsala University Hospital, *n* = 2), Linköping (Linköping University Hospital,* n* = 4), and Lund (Skåne University Hospital, *n* = 8). Healthy pregnant women (HC, *n* = 27) were recruited at one antenatal clinic, Regionhälsan, Gothenburg. All women with SLE fulfilled the 1997 American College of Rheumatology (ACR) and/or the 2012 Systemic Lupus International Collaborating Clinics (SLICC) classification criteria [32, 33]. According to local routine, disease activity was measured at least once between gestational weeks 10 and 34 according to the SLE Disease Activity Index 2000 (SLEDAI-2K) [34]. When multiple measurements were available, a mean SLEDAI-2K value was calculated. Clinical data, including medication, gestational age at birth, and autoantibody positivity were recovered from medical records. Analysis of autoantibodies was performed at each study site and positivity was determined according to cut-off levels at the local laboratories. Exclusion criteria were the inability to understand patient information and treatment with anti-BAFF or anti-CD20 antibodies within 12 months before inclusion. All participants gave written informed consent, and the study was approved by the regional ethics committee of Gothenburg (Dnr 404-18 and amendment Dnr 2020-05101 and Dnr 2022-01158-02) and was conducted in compliance with the Helsinki Declaration.

### Blood samples

Whole blood samples were collected in heparinized tubes in the first, second, and third trimesters (Supplementary Table 1). From 19 of the included women with SLE, an additional blood sample was collected late postpartum (Supplementary Table 1) at least 6 months after delivery (median 10 months, range 6–36 months after delivery). All blood samples were kept at ambient temperature and processed the day after venipuncture within 24 h at our laboratory in Gothenburg.

### Isolation of granulocyte subsets

Granulocyte subsets, i.e., low- and normal-density granulocytes (LDG and NDG) were isolated as previously described in detail [19]. In brief, blood was diluted 1:1 with PBS, layered on Ficoll-Paque plus (GE Healthcare, Uppsala, Sweden), and centrifuged (900 × g, 20 min, without brake). If present, LDG that co-localized with peripheral blood mononuclear cells (PBMCs) were collected from the top of the Ficoll-Paque layer and NDG (pellet) were collected from the bottom of the tube.

### Flow cytometry

The proportion of LDG among PBMCs was analyzed based on conventional gating using CD45 expression and side scatter characteristics. The vast majority of defined LDG in SLE and HC expressed the neutrophil marker CD15 but were negative for the monocyte marker CD14 (Supplementary Fig. 1A). The activation status of LDG and NDG was defined by CD62L shedding. In brief, red blood cells in the pellet were lysed twice by a short incubation with dH_2_O followed by the addition of PBS (with 25 g NaCl/L). NDG and PBMCs including LDG were stained with antibodies against CD45 and CD62L. In a subgroup of pregnant women with SLE and healthy pregnant controls, not included in Table 1, LDG and NDG were stained with CD10 to identify mature and immature LDG and NDG. All antibodies used are presented in Supplementary Table 2. To exclude dead cells, 7-aminoactinomycin D (7AAD, BD Biosciences) was used. TruCount™ assay was used to analyze the total number of granulocytes in whole blood. Red blood cells were lysed using FACS™ Lysing solution (BD Biosciences). Same day processing of blood was not feasible due to sample transportation from distant study sites to our laboratory, but we found no difference in LDG proportions within the time span when all samples in the study were analyzed (i.e., between 17 and 24 h post venipuncture) in either SLE or HC, or when comparing the processing of blood from one pregnant woman with SLE or one pregnant HC at 5 compared to 24 h (Supplementary Fig. 1B–C). Regarding CD62L shedding, there was no difference between 17 and 24 h after sampling and a 2.3-fold (SLE) and 4.8-fold (HC) increase between 5 and 24 h post venipuncture (Supplementary Fig. 1B–C). Thus, as all samples were analyzed between 17 and 24 h after sampling, we consider granulocyte activation status comparable between the two groups. All samples were acquired in a FACSVerse equipped with FACSuite Software (BD Biosciences) and analyzed with FlowJo Software (TreeStar, Ashland, Oregon, USA).

**Table 1:** Characteristics of women with SLE and healthy controls

	Women with SLE (*n* = 69)	Healthy women (*n* = 27)
Age (years), median (range)^a^	32 (23–43)	29 (24–41)
Nulliparous, *n* (%)	41 (59)	21 (78)
Disease duration at inclusion (years), median (range)	9 (0–26)	
SLEDAI-2K during pregnancy, median (range)^b^	1 (0–14)	
ACR criteria ever, *n* (%)		
Malar Rash	28 (41)	
Discoid rash	4 (6)	
Photosensitivity	35 (51)	
Oral ulcers	28 (41)	
Arthritis	58 (84)	
Serositis	12 (17)	
Renal disorder	26 (38)	
Neurological disorder	4 (6)	
Hematological disorder	41 (59)	
Immunological disorder	59 (86)	
ANA	68 (99)	
Autoantibodies ever, *n* (%)		
Anti-dsDNA	58 (84)	
Anti-Sm^c^	19 (28)	
Anti-SSA	22 (32)	
Anti-SSB	12 (17)	
Lupus anticoagulant	11 (16)	
Anti-cardiolipin IgG	10 (15)	
Anti-β2glycoprotein I IgG^c^	11 (16)	
Ever antiphospholipid antibodies	17 (25)	
Antiphospholipid syndrome, *n* (%)	4 (6)	
Medication in early pregnancy, *n* (%)		
Hydroxychloroquine or chloroquine phosphate	63 (91)	
Acetylsalicylic acid	60 (87)	
Low molecular weight heparin	14 (20)	
Azathioprine	20 (29)	
Prednisone	18 (26)	

^a^Age of mother at gestational week 12

^b^Missing data from five patients

^c^Missing data from one patient

### Quantification of IFNα, G-CSF, and GM-CSF

Plasma was kept frozen at −80 °C until analysis. IFNα protein concentration was measured with single molecule array (Simoa) digital ELISA on an HD-X Analyzer (Quanterix, Billerica, MA). The Simoa assay contained an inhibitor for heterophilic antibodies to prevent false-positive results. The lower limit of quantification was 70 fg/ml. In cases in which a sample was below the lower limit of quantification, its value was adjusted to 35 fg/ml when used in analysis. IFNα protein positivity was defined as a level ≥ 136 fg/ml based on three standard deviations above the mean level for healthy blood donors as previously measured using the same method [24]. The concentration of granulocyte colony-stimulating factor (G-CSF) and granulocyte-macrophage colony-stimulating factor (GM-CSF) were analyzed by bead-based immunoassay (LEGENDplex™ Human Growth Factor Panel, BioLegend, San Diego, CA) according to the manufacturer’s instructions, and acquired on a FACSVerse. Data was analyzed using FlowJo Software. The lower limit of quantification was 48.8 pg/ml for both proteins. In cases in which a sample was below the lower limit of quantification, its value was adjusted to 24.4 pg/ml when used in analysis.

### Statistical analysis

Multivariate data analysis was performed using SIMCA-P software version 13.0.3 (Sartorius Stedim Biotech, Goettingen, Germany). Principal Component Analysis (PCA) was performed to make an unsupervised analysis and to visualize the relationship between LDG proportions, NDG activation status, IFNα protein concentrations, autoantibody profile, and gestational age at birth in SLE pregnancy. Orthogonal partial least squares analysis (OPLS) was implemented to examine gestational age at birth or specific drug treatments (Y-variables) in relation to immune variables (X-variables). Default settings for the PCA and OPLS models were used; data were centered and scaled to unit-variance (UV) in the software to give all variables equal weight. Model quality was based on R2 and Q2 parameters and are presented in each figure. Univariate analyses were exclusively performed for Y- and X-variables that showed the strongest associations in the respective models. Univariate analyses performed were Kruskal-Wallis followed by Dunn’s multiple comparison test, Mann-Whitney *U* test, and Spearman rank correlation test (GraphPad prism software, La Jolla, CA, USA) as described in the respective figure legends. Multivariable linear regression analysis was performed using SPSS Statistics 29 (IBM, NY, USA). *P*-values of  < 0.05 were considered statistically significant.

## Results

### Characteristics of pregnant women with SLE and healthy pregnant women

In both groups, the majority of the women were nulliparous (Table 1). The median disease duration was 9 years and all except one were antinuclear antibody (ANA) positive. Most women with SLE were anti-dsDNA positive, approximately one third were anti-Sm and anti-SSA positive, a quarter were antiphospholipid antibody positive (lupus anticoagulant 16%, anti-cardiolipin 15% and anti-β2glycoprotein I 16%) and four had antiphospholipid syndrome. Most women with SLE were treated with hydroxychloroquine and acetylsalicylic acid, while 20% were treated with low molecular weight heparin. Twenty-eight percent of the patients were treated with azathioprine, 26% were treated with prednisone, and 10% of patients were treated with both. Most SLE women had low disease activity, with a median disease activity according to SLEDAI-2K of one. None of the components that contributed to the score were caused by the pregnancy per se but were clearly related to SLE.

### Higher LDG proportions and increased granulocyte activation in blood among pregnant women with SLE

**Fig. 1:**
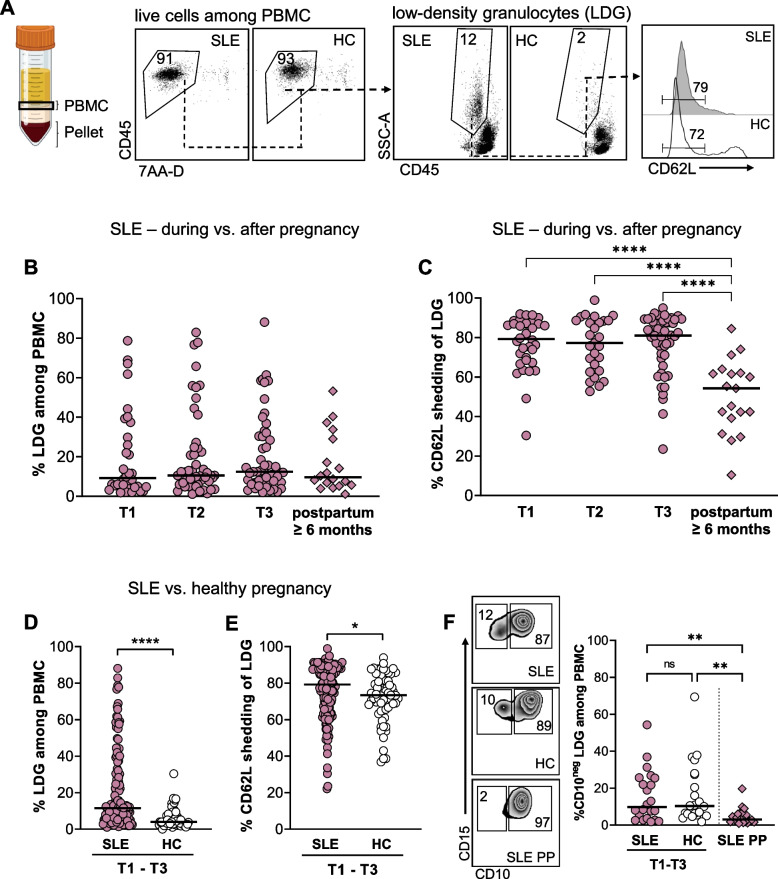
Lupus pregnancy leads to increased LDG activation in blood. **A** Representative flow cytometry plots from SLE and HC that illustrate the gating strategy for proportions of CD62L-negative LDG analyzed among PBMC after density centrifugation. Proportions of LDG (**B**) and proportions of LDG that have shed CD62L (**C**) in the first, second, and third trimesters (T1, T2, and T3) compared to late postpartum in women with SLE. Proportions of LDG (**D**) and proportions of LDG that have shed CD62L (**E**) in pregnant women with SLE compared to pregnant healthy women (combined data from trimesters one to three). **F** Representative flow cytometry plots regarding CD10 expression on LDG from SLE and HC during pregnancy and SLE postpartum, and comparison of CD10-negative immature LDG in SLE and HC during pregnancy and to postpartum in SLE. *p < 0.05, **p < 0.01, and **** *p* < 0.0001, **B**, **C**, and **F** Kruskal-Wallis followed by Dunn’s multiple comparison test and **D** and **E** Mann-Whitney *U* test. Illustration created in BioRender.com

Previous studies have demonstrated increased proportions of LDG in non-pregnant patients with SLE compared to healthy controls [15, 16, 35]. We assessed the prevalence of LDG as well as LDG activation status in our longitudinal cohort of pregnant women with SLE and pregnant HC. A representative gating strategy of LDG in SLE and HC is depicted in Fig. 1A. The PBMC fraction comprised mainly live cells. LDG activation status was defined by CD62L expression as this adhesion molecule is shed from granulocytes upon activation. In most HC, very few LDG were identified and there was no clear LDG population as compared to in SLE. To determine whether potential differences in LDG proportions and/or activation status between SLE and HC pregnancies are due to a combined effect of SLE and pregnancy or to SLE per se, late postpartum data from SLE patients were used as a comparator arm. In SLE, LDG proportions did not differ during pregnancy compared to late postpartum (Fig. 1B), but LDG were more activated during compared to after pregnancy (Fig. 1C). This indicates that the presence of LDG is due to SLE and not affected by pregnancy, whereas LDG activation, as seen by CD62L shedding, is a feature associated with pregnancy in SLE. Similar results were observed when only including women from whom postpartum samples were collected (Supplementary Fig. 2A–B). Nine pregnant women with SLE had moderate to high disease activity (SLEDAI-2K ≥ 4) and there was a non-significant trend of higher LDG proportions, mainly in trimester three, among women with moderate-high disease activity compared to those with low activity (Supplementary Fig. 3A–C). Pregnant women with SLE had significantly higher LDG proportions than pregnant HC throughout pregnancy (Fig. 1D) and per trimester (Supplementary Fig. 4A). SLE LDG were slightly more activated than HC LDG (Fig. 1E). When compared per trimester, SLE LDG were significantly more activated relative to HC LDG in trimester one and there was a trend for higher activation in trimester three (Supplementary Fig. 4B). Late postpartum samples from HC were not collected in this cohort, but LDG from pregnant HC appeared to be more activated than LDG from late postpartum in SLE (median CD62L shedding = 73.4% vs median CD62L shedding = 54.3%). Thus, regardless of SLE status, LDG may be more activated during compared to after pregnancy.

To investigate the LDG maturation state, the proportions of CD10-expressing cells were examined in a subgroup of patients with SLE and HC. CD10 is a transmembrane glycoprotein that is expressed by mature granulocytes at their latest stages of differentiation. We found no difference in circulating proportions of immature CD10-negative LDG during pregnancy between SLE and HC, but both SLE and HC had significantly higher proportions of immature LDG during pregnancy compared to late postpartum in SLE (Fig. 1F).

**Fig. 2:**
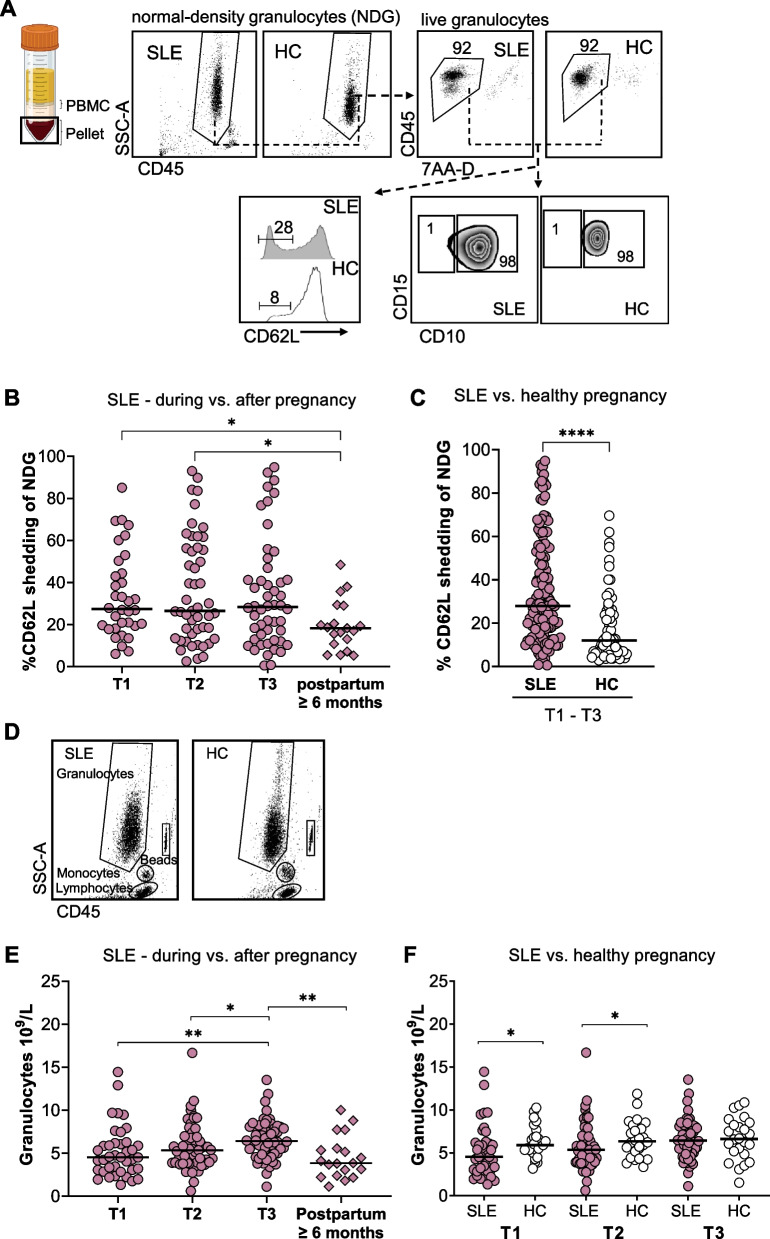
NDG are more activated during SLE compared to a healthy pregnancy. **A** Representative flow cytometry plots from SLE and HC that illustrate the gating strategy for proportions of NDG that have shed CD62L and NDG that express CD10, respectively. Proportions of NDG that have shed CD62L (**B**) in the first, second, and third trimesters (T1, T2, and T3) compared to late postpartum in women with SLE, and (**C**) in SLE compared to HC pregnancies (combined data from trimester one to three). **D** TruCount gating strategy for granulocytes in SLE and healthy women. **E** Total granulocyte counts in blood in the first, second, and third trimesters compared to late postpartum in women with SLE. **F** Total granulocyte counts in the first, second, and third trimesters in pregnant women with SLE compared to pregnant healthy women. *p ≤ 0.05, **p < 0.01 and ****p < 0.0001, **B** and **E** Kruskal-Wallis followed by Dunn’s multiple comparison test and **C** and **F** Mann-Whitney *U* test. Illustration created in BioRender.com

Next, we examined the NDG activation status. A representative gating strategy in SLE and HC is presented in Fig. 2A, which shows that the vast majority of analyzed cells were live NDG. In both SLE and HC pregnancies, almost all NDG were mature CD10-positive cells (Fig. 2A). Like LDG, SLE NDG were more activated during pregnancy compared to late postpartum (Fig. 2B and Supplementary Fig. 2C), and SLE NDG were more activated than HC NDG throughout pregnancy (Fig. 2C) and per trimester (Supplementary Fig. 4C). In both SLE and HC pregnancies, circulating NDG were less activated than LDG (Supplementary Fig. 5).

Total granulocyte count in whole blood was measured based on CD45 expression and side scatter characteristics (Fig. 2D). In SLE pregnancies, total granulocyte numbers increased from trimester one to three and thereafter decreased in the late postpartum period (Fig. 2E and Supplementary Fig. 2D). Women with SLE displayed lower granulocyte numbers in the first and second trimester relative to HC (Fig. 2F). Both G-CSF and GM-CSF are important for granulopoiesis, but we found no differences in the levels of either protein during pregnancy compared to late postpartum in SLE, between trimesters in SLE, or between SLE and HC (Supplementary Fig. 6A–D). Moreover, treatment with prednisone, azathioprine, or heparin showed no clear effect on LDG proportions, granulocyte activation/numbers, or G-CSF/GM-CSF protein levels (Supplementary Fig. 7A–C). The effect of hydroxychloroquine or acetylsalicylic acid could not be analyzed as most patients had these treatments. In summary, SLE pregnancy results in augmented activation of both LDG and NDG and an increase in granulocyte numbers in blood relative to late postpartum. Women with SLE also display increased LDG proportions and increased granulocyte activation throughout pregnancy compared to healthy women.

### IFNα protein positivity is present in a subgroup of pregnant women with SLE

**Fig. 3:**
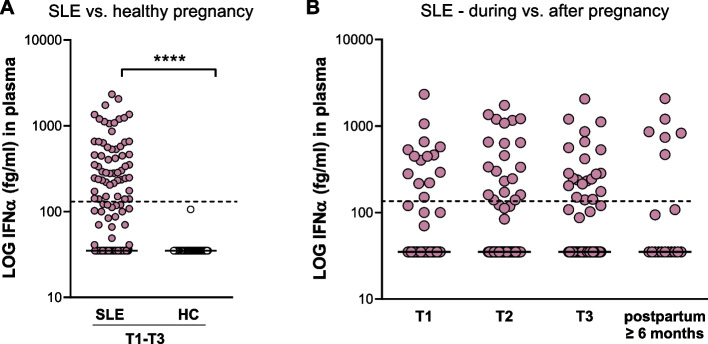
IFNα protein positivity is present in a subgroup of women with SLE. **A** Comparison of IFNα protein concentrations in plasma between pregnant women with SLE and healthy pregnant HC (combined data from trimesters one to three). **B** Plasma IFNα protein concentrations in the first, second, and third trimesters compared to late postpartum in women with SLE. The dotted line denotes the cut-off for IFNα positivity (136 fg/mL). **** *p* < 0.0001, Mann-Whitney *U* test

Given the reports that SLE LDG spontaneously form NETs, a potential stimuli for increased IFNα production by pDCs [17, 28], we measured IFNα protein concentrations in our cohort. Women with SLE had higher IFNα levels compared to HC throughout pregnancy (Fig. 3A) and per trimester (Supplementary Fig. 4D). Among women with SLE, 36% were IFNα protein-positive in comparison with none of the pregnant HC. IFNα concentrations did not differ between trimesters or compared to late postpartum in SLE (Fig. 3B). IFNα concentrations were unrelated to treatment with prednisone, azathioprine, or heparin (Supplementary Fig. 8). These results indicate that IFNα plasma protein concentrations are due to the disease and not affected by pregnancy in SLE.

### LDG proportions and granulocyte activation are associated with antiphospholipid antibody positivity but unrelated to IFNα protein levels in SLE pregnancy

**Fig. 4:**
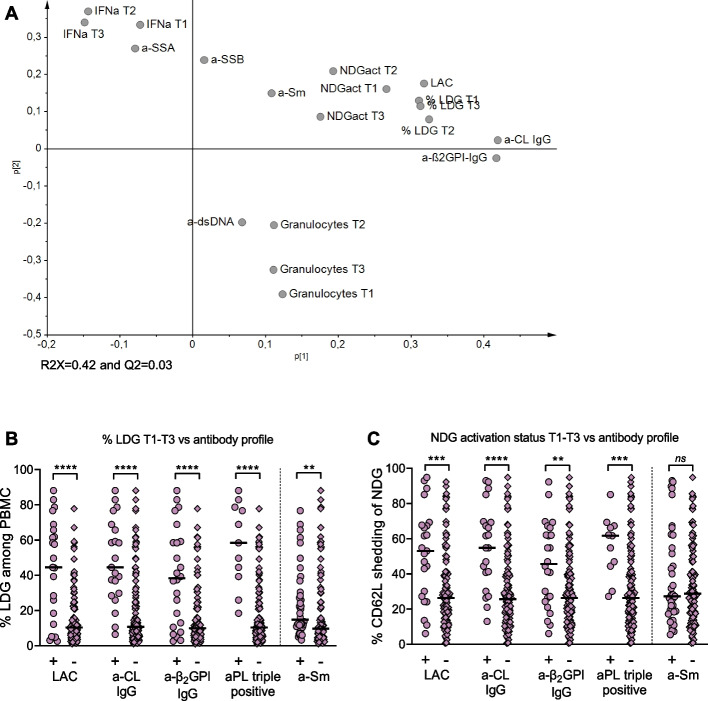
LDG proportions and NDG activation relate to aPL positivity but not to IFNα in SLE. **A** Unsupervised principal component analysis demonstrating the relationship between LDG proportions, NDG activation, IFNα protein concentrations, specific autoantibody positivity, and total granulocyte numbers in SLE pregnancies. The frequency of LDG (**B**) or NDG activation status (**C**) in pregnant SLE women stratified according to an antiphospholipid antibody or anti-Sm serologic profile. **p < 0.01, ***p < 0.001 and ****p < 0.0001, Mann-Whitney *U* test

Higher LDG proportions have been demonstrated in non-pregnant SLE patients presenting with a high compared to a low IFN gene signature [16, 30], and in non-pregnant SLE patients with antiphospholipid antibodies (aPL) compared to those without [31]. Using multivariate principal component analysis (PCA), we investigated whether LDG proportions and NDG activation status relate to IFNα protein concentrations and/or specific autoantibody positivity in pregnant women with SLE. The PCA plot in Fig. 4A shows that IFNα protein levels are projected in the upper left quadrant together with anti-SSA positivity. IFNα levels were not associated with LDG fractions or NDG activation as these variables were projected in the upper right quadrant together with aPL-positivity including, lupus anticoagulant (LAC), anti-β2glycoprotein I (β_2_GPI) and anti-cardiolipin (CL), as well as with anti-Sm positivity. Finally, total granulocyte numbers were clustered with anti-dsDNA positivity in the lower right quadrant. In univariate analysis, IFNα levels were higher in anti-SSA positive women compared to those who were negative (Supplementary Fig. 9), but unrelated to LDG proportions (*r* = 0.08 and *p* = 0.38). LDG proportions and NDG activation status were higher in women who were positive for LAC, anti-CL IgG, and anti-β_2_GPI IgG or were triple positive compared to women negative for the respective antibodies (Fig. 4B–C). Anti-Sm positivity was related to higher LDG proportions, but not to NDG activation (Fig. 4B–C). Finally, there was no difference in total granulocyte numbers in anti-dsDNA positive compared to negative women (*p* = 0.39). Thus, higher circulating LDG proportions are related to aPL positivity but not to IFNα protein concentrations in SLE pregnancies.

### LDG proportions are related to shorter pregnancy duration in SLE

**Fig. 5:**
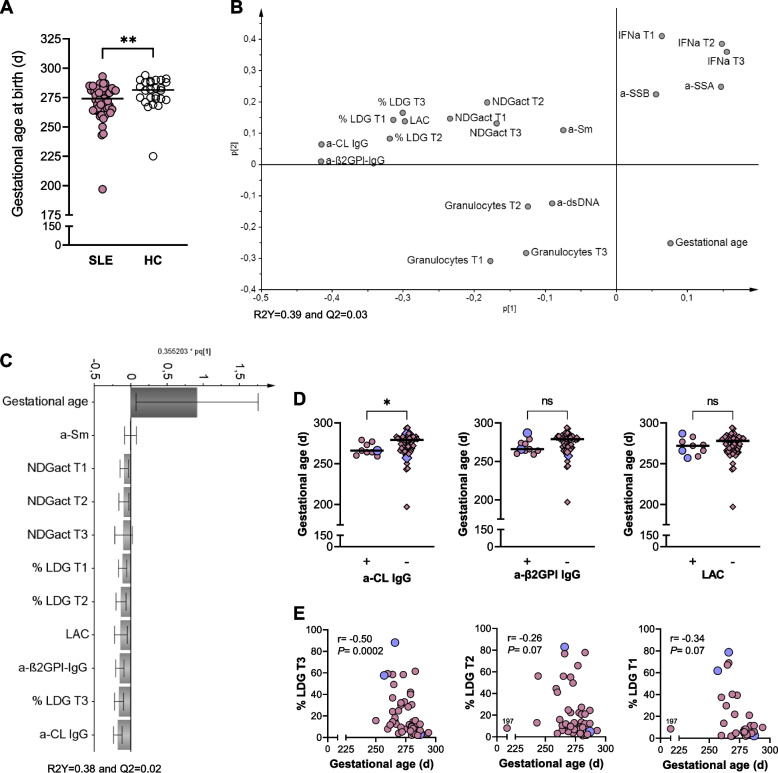
Higher LDG proportions correlate with lower gestational age at birth in SLE. **A** Gestational age at birth in SLE compared to healthy pregnancies. **B** Unsupervised principal component analysis (PCA) demonstrating the relationship between gestational age at birth, LDG proportions, NDG activation, IFNα protein concentrations, specific autoantibody positivity, and total granulocyte numbers in SLE pregnancies. **C** OPLS plot depicting gestational age at birth in relation to negatively related variables identified in PCA analysis. **D** Gestational age at birth in women with SLE who were positive for anti-CL, anti-β2GPI, or LAC compared to those who were negative for the respective antibodies. **E** Correlations between gestational age at birth and proportions of LDG in trimesters three, two, or one, respectively, in SLE. Subjects with SLE and antiphospholipid syndrome are indicated by large blue symbols in **D** and **E**. *p < 0.05 and **p < 0.01, **A** and **D** Mann-Whitney *U* test and **E** Spearman rank correlation test

Given that pregnant women with SLE face an increased risk of preterm delivery, unsupervised PCA was performed to examine whether gestational age at birth (GA) was related to LDG proportions, granulocyte activation and numbers, IFNα protein levels or specific autoantibody profile in SLE. Women with SLE displayed a lower GA compared to healthy women (median 274 days for SLE and 282 days for HC, Fig. 5A). In the PCA plot, GA was the only variable projected in the lower right quadrant, indicating that none of the immunological variables were positively related to higher GA (Fig. 5B). Inversely related to GA were aPL positivity, LDG proportions, and granulocyte activation as these variables were projected in the upper left quadrant, while IFNα protein levels, a-SSA, a-SSB, a-dsDNA, and granulocyte numbers showed no association with GA (Fig. 5B). Next, OPLS analysis was used to investigate GA in relation to inversely associated variables. As shown in Fig. 5C, GA was most negatively associated with anti-CL IgG, LDG proportions in trimester 3, and anti-β2GPI IgG. In univariate analysis we found that anti-CL-positive women had lower GA compared to those who were negative, while there were no significant differences in GA between those who were positive versus negative for anti-β2GPI IgG or LAC (Fig. 5D). We also found a significant inverse correlation between GA and LDG proportions in trimester three but not in trimester two and one (Fig. 5E). Moreover, similar results were observed when GA was stratified into spontaneous or induced delivery among women with SLE (Supplementary Fig. 10). Finally, multivariable linear regression analysis showed that higher LDG proportions in trimester three were associated with lower GA independently of anti-CL IgG positivity (LDG T3 B =  − 0.368, *p* = 0.02 and anti-CL IgG B =  − 0.098, *p* = 0.52). Thus, higher proportions of LDG in the blood are related to shorter pregnancy duration independently of aPL positivity among women with SLE.

## Discussion

Although knowledge about SLE-related pregnancy risk factors and treatment has improved, women with SLE still face an increased risk of pregnancy complications compared to the general population [2, 4]. Thus, identification of specific immunopathological mechanisms that can lead to complications is needed to further improve outcomes and to predict which women are at the highest risk of developing them. Elevated LDG proportions, increased granulocyte activation, and type I IFN overproduction are common features in SLE [13, 16, 23, 35], and aPL positivity is a risk factor for pregnancy complications in SLE [4]. However, the relationship between these immunopathologic disease-related features has not previously been investigated in SLE pregnancy. We here demonstrate that lupus pregnancy resulted in increased granulocyte activation compared to postpartum and that the activation status was higher in SLE compared to in HC. Moreover, women with SLE presented with higher LDG fractions as well as IFNα protein levels compared to HC throughout pregnancy, but these factors were not related to each other. Finally, LDG proportions in trimester three and anti-CL positivity were associated with lower gestational age at birth, but higher LDG fractions contributed independently of aPL positivity to shorter pregnancy duration in SLE.

We recently showed that women with SLE have higher proportions of LDG in blood relative to healthy women at delivery [19], and in this longitudinal study, we demonstrate that LDG are elevated throughout pregnancy in SLE. Even though pregnancy had no effect on LDG frequencies in SLE, both LDG and NDG were more activated during pregnancy compared to late postpartum in SLE women. Furthermore, both granulocyte subsets, NDG in particular, were more activated in SLE compared to HC pregnancies. We found no association between the presence of LDG or granulocyte activation status and medication use, including prednisone and azathioprine. Our results are in accordance with previous studies that reported higher LDG proportions and/or counts in non-pregnant patients with SLE compared to controls, which was unrelated to the use of immunosuppressive drugs [16, 35].

LDG are considered as contributors to the IFN signature in SLE due to their spontaneous ability to form NETs that stimulate pDCs to secrete IFNα in vitro [17, 28, 35, 36]. In support of this, LDG proportions are higher in SLE patients with a high IFN signature compared to those with a low signature and in SLE patients with moderate/high disease activity (SLEDAI ≥ 4) relative to those with low disease activity [16, 37]. For the first time we here measured IFNα protein blood levels in SLE pregnancies. Even if most women with SLE had hydroxychloroquine treatment (91%), which is known to decrease the IFN signature strength and to reduce IFNα secretion by pDCs [38, 39], we found that about one third were IFNα protein positive. In contrast to what has been shown for the IFN signature, we found IFNα protein levels, primarily IFNα2, to be unrelated to LDG proportions. The reason for this discrepancy could be due to that additional factors than IFNα affect the IFN signature such as other interferons and/or yet unidentified proteins [40]. Indeed, different modules of IFN-regulated genes respond not only to IFNα but also to IFNβ and IFNγ [41, 42].

An association between high IFNα blood levels and anti-SSA autoantibody positivity has previously been shown in SLE [40]. In line with this, we found higher IFNα protein levels in anti-SSA positive compared to anti-SSA negative pregnant women with SLE. This relationship was also recently described in non-pregnant patients with primary Sjögren’s syndrome using the same technology to measure IFNα [43]. Higher LDG proportions were related to aPL positivity in our study of pregnant women, which has previously been described in non-pregnant patients with SLE [31]. In addition, aPL positivity was also related to higher NDG activation status in SLE pregnancies. Whether there is a causality between aPL, higher LDG proportions and NDG activation is not answered by the present data, but in vitro studies indicate that aPL prime/activate neutrophils [44].

Higher proportions of LDG correlated with shorter pregnancy duration in SLE. The reason for this could only be speculated upon, but experimental studies suggest that SLE LDG are retained in microvasculature networks like primed NDG from healthy donors [45], and NET-forming LDG are associated with endothelial cell damage [12] and vascular inflammation in SLE [13]. The placenta is a highly vascularized organ and circulating activated LDG may contribute to placental inflammation and dysfunction. Indeed, more NETs are found in the intervillous space in the placentas of women with SLE compared to healthy women [46]. We also found that anti-CL positive women had shorter pregnancy duration compared to negative women. In line with this, aPL positivity is a predictor for poor fetal outcomes, including prematurity and intrauterine growth restriction [10]. In multivariate regression analysis higher LDG proportions in trimester three contributed independently of anti-CL positivity to shorter pregnancy duration in our cohort.

There are different theories about the LDG origin and whether they represent immature cells, mature and activated cells, or a mix [18, 47]. Here, we found a higher proportion of immature CD10-negative LDG in blood during pregnancy among both SLE and HC when compared to the late postpartum period in SLE where almost all LDG consisted of mature CD10-positive cells. Neutrophil maturation in pregnancy has previously been examined in a small cohort of healthy women using mass cytometry [48]. In line with our results, they found a decreased intensity in CD10 expression on total neutrophils in late pregnancy compared to 6 weeks postpartum [48]. The reason for a higher proportion of immature granulocytes in blood during pregnancy is not known, but is likely explained by an increased release of more immature granulocytes from the bone marrow. Accordingly, the total granulocyte number in the blood is increased in pregnant compared to non-pregnant healthy women [49, 50]. This phenomenon was also observed among women with SLE in the present study, even if granulocyte numbers were lower in SLE relative to HC in trimesters one and two. Both G-CSF and GM-CSF are important for granulopoiesis, and the concentrations of these proteins are increased in pregnant compared to non-pregnant healthy women [49, 51]. However, G-CSF and GM-CSF could not explain differences in granulocyte numbers in our study as we found no differences in levels of these proteins during pregnancy compared to late postpartum in SLE, between trimesters in SLE, or between SLE and HC.

The use of fresh samples from well-characterized patients, collected prospectively during pregnancy and in the late postpartum period, should be considered a key strength of this study. Secondly, women with SLE and HC were included into the study in parallel. Lastly, all immunologic assays were performed and analyzed in one laboratory. A limitation is that our study includes missing data, mainly from the first trimester, among pregnant women with SLE. Another is that our cohort had very few women with moderate or high SLE disease activity, and therefore our results only reflect a well-controlled cohort of pregnant women with SLE.

In conclusion, our study identifies significant modulation of granulocyte activation during SLE pregnancy and shows that higher LDG proportions in trimester three correlate with shorter pregnancy duration in SLE independently of aPL positivity. A deeper understanding of increased LDG in SLE as well as the immunological mechanisms that lead to peripheral granulocyte priming in lupus pregnancies may result in the development of novel strategies to reduce the risk of unfavorable pregnancy outcomes in women with SLE.

## Supplementary Information

**Additional file 1: Supplementary figure 1.** (A) Expression of CD15 and CD14 on low-density granulocytes (LDG) from two pregnant women with SLE (two left panels, samples from trimester three) and two healthy pregnant controls (HC, two right panels, one sample from trimester three and one sample from trimester one). (B) Analysis of proportions of LDG and shedding of CD62L by NDG in blood from pregnant women with SLE and healthy pregnant controls between 17 and 24 h post venipuncture i.e., the time span when all samples in the study were analyzed. (C) Analysis of total number of granulocytes, proportions of LDG and shedding of CD62L by normal-density granulocytes (NDG) from one pregnant woman with SLE in trimester three and one healthy pregnant control in trimester one at 5 and 24 h post venipuncture. **Supplementary figure 2.** Comparison of (A) proportions of low-density granulocytes (LDG), (B) proportions of LDG that have shed CD62L, (C) proportions of normal-density granulocytes (NDG) that have shed CD62L and (D) total granulocyte counts during compared to after pregnancy among women with SLE from whom late postpartum samples were collected. *p < 0.05, **p < 0.01 and ***p < 0.001, Kruskal-Wallis followed by Dunn’s multiple comparison test. **Supplementary figure 3.** Comparison of LDG proportions in pregnant women with SLE with or without a moderate/high disease activity (SLEDAI-2K ≥ 4) in (A) trimester one, (B) trimester two and (C) trimester three. Mann-Whitney U test. **Supplementary figure 4.** Comparison of (A) LDG proportions, (B) LDG activation by CD62L shedding, (C) NDG activation by CD62L shedding and (D) IFNα protein levels in SLE and healthy pregnancy for each trimester. *p < 0.05, **p < 0.01, ***p < 0.001, ****p < 0.0001 Mann-Whitney U test. **Supplementary figure 5.** Comparison of the proportions of low-density granulocytes (LDG) and normal-density granulocytes (NDG) that have shed CD62L in pregnant women with SLE and in healthy pregnant controls (HC). **** *p* < 0.0001, Mann-Whitney U test. **Supplementary figure 6.** Concentrations of G-CSF (A) and GM-CSF (B) in plasma during pregnancy (first, second and third trimester) compared to the late postpartum period among women with SLE from whom late postpartum samples were collected. Concentrations of G-CSF (C) and GM-CSF (D) in plasma in the first, second and third trimester in SLE compared to healthy pregnancies. **Supplementary figure 7.** (A) OPLS loading column plot depicting granulocyte-related immune variables positively or negatively associated with prednisone use in SLE pregnancy, and low-density proportions in pregnant women with SLE who were treated with prednisone compared to those who were not treated. (B) OPLS plot depicting granulocyte-related immune variables positively or negatively associated with azathioprine use in SLE pregnancy and CD62L shedding of normal-density granulocytes in pregnant women with SLE who were treated with azathioprine compared to those who were not treated. (C) OPLS plot depicting granulocyte-related immune variables positively or negatively associated with low molecular weight heparin use in SLE pregnancy. * *p* < 0.05, Mann-Whitney U test. **Supplementary figure 8.** IFNα plasma protein concentrations in trimester one, two and three from pregnant women with SLE who were treated or not with prednisone (A), azathioprine (B) or low molecular weight heparin (C). **Supplementary figure 9.** IFNα plasma protein concentrations in trimester one, two and three from pregnant women with SLE who were anti-SSA positive compared to those who were negative. ** *p* < 0.01, Mann-Whitney U test. **Supplementary figure 10.** (A) Gestational age at birth among women with SLE with induced compared to spontaneous delivery. (B) Gestational age at birth in women with SLE with induced or spontaneous delivery who were positive or negative for anti-CL, anti-β2GPI or LAC. (C) Correlations between gestational age at birth and proportions of LDG in trimester three, two and one among women with SLE with induced or spontaneous delivery. * *p* < 0.05, (B) Mann-Whitney U test and (C) Spearman rank correlation test.

**Additional file 2: Supplementary Table 1.** Numbers of collected blood samples. **Supplementary Table 2.** Antibodies used for flow cytometry.

## Data Availability

Data that was used in this study are available from the corresponding author Agnes Torell or Anna-Carin Lundell upon reasonable request.

## Abbreviations

ACR: American College of Rheumatology
ANA: Antinuclear antibodies
aPL: Antiphospholipid antibodies
ELISA: Enzyme-linked immunosorbent assay
GA: Gestational age
G-CSF: Granulocyte colony-stimulating factor
GM-CSF: Granulocyte-macrophage colony-stimulating factor
HC: Healthy controls
IFN: Interferon
LAC: Lupus anticoagulant
LDG: Low-density granulocyte(s)
NDG: Normal-density granulocyte(s)
NET: Neutrophil extracellular trap
OPLS: Orthogonal projection to latent structures
PBMC: Peripheral blood mononuclear cells
PCA: Principal component analysis
pDCs: Plasmacytoid dendritic cells
Simoa: Single molecular array
SLE: Systemic lupus erythematosus
SLICC: Systemic Lupus International Collaborating Clinics
β_2_GPI: β_2_ Glycoprotein I
