# Supplementary material for: Low-density granulocytes are related to shorter pregnancy duration but not to interferon alpha protein blood levels in systemic lupus erythematosus
Source: Arthritis Res Ther. 2023 Jun 22;25:107. doi: 10.1186/s13075-023-03092-w (PMC10286457; doi:10.1186/s13075-023-03092-w)
Supplement: Supplementary file 1 — Additional file 1: Supplementary figure 1. (A) Expression of CD15 and CD14 on low-density granulocytes (LDG) from two pregnant women with SLE (two left panels, samples from trimester three) and two healthy pregnant controls (HC, two right panels, one sample from trimester three and one sample from trimester one). (B) Analysis of proportions of LDG and shedding of CD62L by NDG in blood from pregnant women with SLE and healthy pregnant controls between 17 and 24 h post venipuncture i.e., the time span when all samples in the study were analyzed. (C) Analysis of total number of granulocytes, proportions of LDG and shedding of CD62L by normal-density granulocytes (NDG) from one pregnant woman with SLE in trimester three and one healthy pregnant control in trimester one at 5 and 24 h post venipuncture. Supplementary figure 2. Comparison of (A) proportions of low-density granulocytes (LDG), (B) proportions of LDG that have shed CD62L, (C) proportions of normal-density granulocytes (NDG) that have shed CD62L and (D) total granulocyte counts during compared to after pregnancy among women with SLE from whom late postpartum samples were collected. *p < 0.05, **p < 0.01 and ***p < 0.001, Kruskal-Wallis followed by Dunn’s multiple comparison test. Supplementary figure 3. Comparison of LDG proportions in pregnant women with SLE with or without a moderate/high disease activity (SLEDAI-2K ≥ 4) in (A) trimester one, (B) trimester two and (C) trimester three. Mann-Whitney U test. Supplementary figure 4. Comparison of (A) LDG proportions, (B) LDG activation by CD62L shedding, (C) NDG activation by CD62L shedding and (D) IFNα protein levels in SLE and healthy pregnancy for each trimester. *p < 0.05, **p < 0.01, ***p < 0.001, ****p < 0.0001 Mann-Whitney U test. Supplementary figure 5. Comparison of the proportions of low-density granulocytes (LDG) and normal-density granulocytes (NDG) that have shed CD62L in pregnant women with SLE and in healthy pregnant controls (HC). **** p < 0.0001 [file 13075_2023_3092_MOESM1_ESM.pptx]

## Slide 1
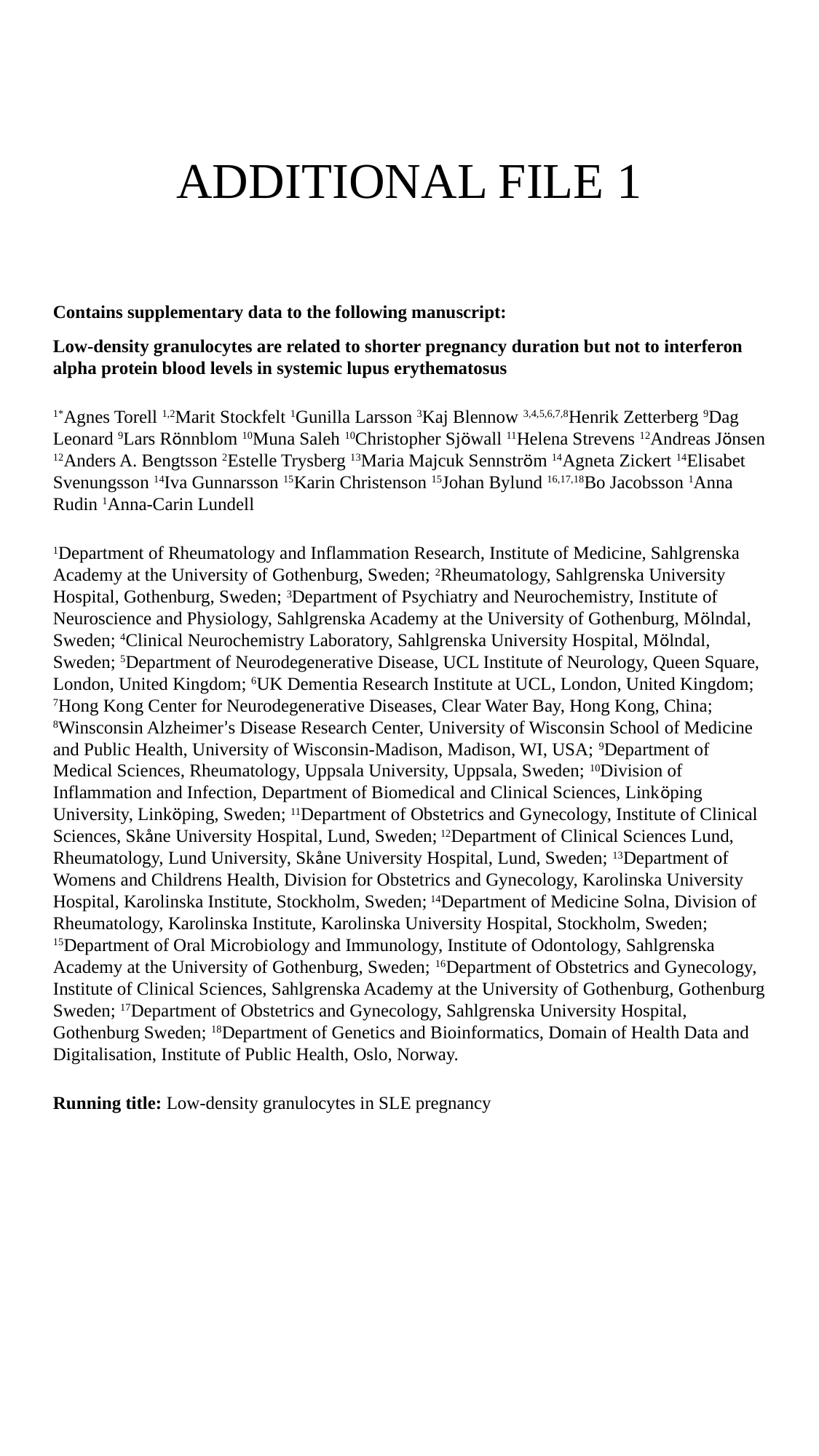

ADDITIONAL FILE 1
Contains supplementary data to the following manuscript:
Low-density granulocytes are related to shorter pregnancy duration but not to interferon alpha protein blood levels in systemic lupus erythematosus
1*Agnes Torell 1,2Marit Stockfelt 1Gunilla Larsson 3Kaj Blennow 3,4,5,6,7,8Henrik Zetterberg 9Dag Leonard 9Lars Rönnblom 10Muna Saleh 10Christopher Sjöwall 11Helena Strevens 12Andreas Jönsen 12Anders A. Bengtsson 2Estelle Trysberg 13Maria Majcuk Sennström 14Agneta Zickert 14Elisabet Svenungsson 14Iva Gunnarsson 15Karin Christenson 15Johan Bylund 16,17,18Bo Jacobsson 1Anna Rudin 1Anna-Carin Lundell
1Department of Rheumatology and Inflammation Research, Institute of Medicine, Sahlgrenska Academy at the University of Gothenburg, Sweden; 2Rheumatology, Sahlgrenska University Hospital, Gothenburg, Sweden; 3Department of Psychiatry and Neurochemistry, Institute of Neuroscience and Physiology, Sahlgrenska Academy at the University of Gothenburg, Mölndal, Sweden; 4Clinical Neurochemistry Laboratory, Sahlgrenska University Hospital, Mölndal, Sweden; 5Department of Neurodegenerative Disease, UCL Institute of Neurology, Queen Square, London, United Kingdom; 6UK Dementia Research Institute at UCL, London, United Kingdom; 7Hong Kong Center for Neurodegenerative Diseases, Clear Water Bay, Hong Kong, China; 8Winsconsin Alzheimer’s Disease Research Center, University of Wisconsin School of Medicine and Public Health, University of Wisconsin-Madison, Madison, WI, USA; 9Department of Medical Sciences, Rheumatology, Uppsala University, Uppsala, Sweden; 10Division of Inflammation and Infection, Department of Biomedical and Clinical Sciences, Linköping University, Linköping, Sweden; 11Department of Obstetrics and Gynecology, Institute of Clinical Sciences, Skåne University Hospital, Lund, Sweden; 12Department of Clinical Sciences Lund, Rheumatology, Lund University, Skåne University Hospital, Lund, Sweden; 13Department of Womens and Childrens Health, Division for Obstetrics and Gynecology, Karolinska University Hospital, Karolinska Institute, Stockholm, Sweden; 14Department of Medicine Solna, Division of Rheumatology, Karolinska Institute, Karolinska University Hospital, Stockholm, Sweden; 15Department of Oral Microbiology and Immunology, Institute of Odontology, Sahlgrenska Academy at the University of Gothenburg, Sweden; 16Department of Obstetrics and Gynecology, Institute of Clinical Sciences, Sahlgrenska Academy at the University of Gothenburg, Gothenburg Sweden; 17Department of Obstetrics and Gynecology, Sahlgrenska University Hospital, Gothenburg Sweden; 18Department of Genetics and Bioinformatics, Domain of Health Data and Digitalisation, Institute of Public Health, Oslo, Norway.
Running title: Low-density granulocytes in SLE pregnancy

## Slide 2
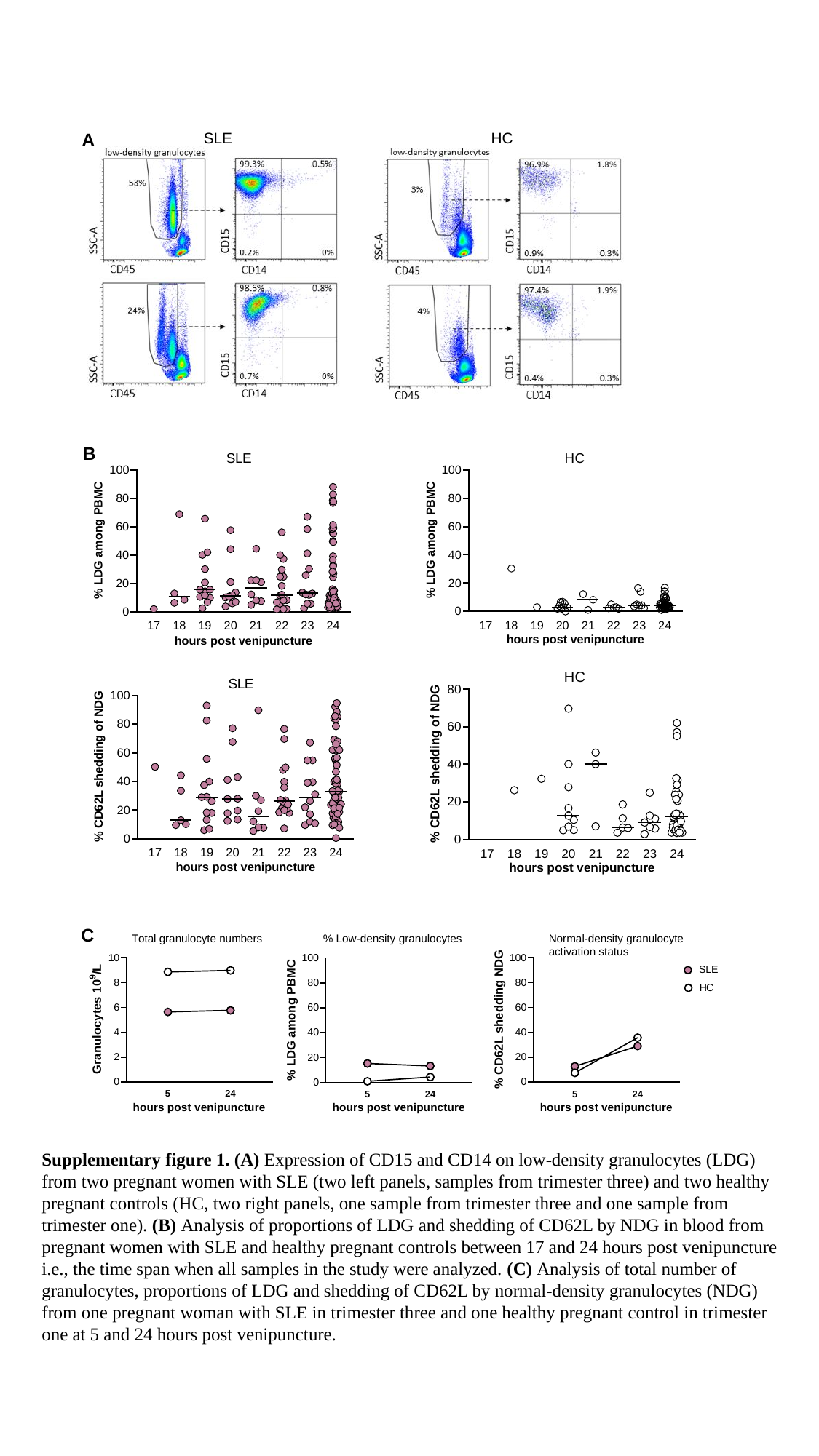

A
SLE
HC
B
C
Supplementary figure 1. (A) Expression of CD15 and CD14 on low-density granulocytes (LDG) from two pregnant women with SLE (two left panels, samples from trimester three) and two healthy pregnant controls (HC, two right panels, one sample from trimester three and one sample from trimester one). (B) Analysis of proportions of LDG and shedding of CD62L by NDG in blood from pregnant women with SLE and healthy pregnant controls between 17 and 24 hours post venipuncture i.e., the time span when all samples in the study were analyzed. (C) Analysis of total number of granulocytes, proportions of LDG and shedding of CD62L by normal-density granulocytes (NDG) from one pregnant woman with SLE in trimester three and one healthy pregnant control in trimester one at 5 and 24 hours post venipuncture.

## Slide 3
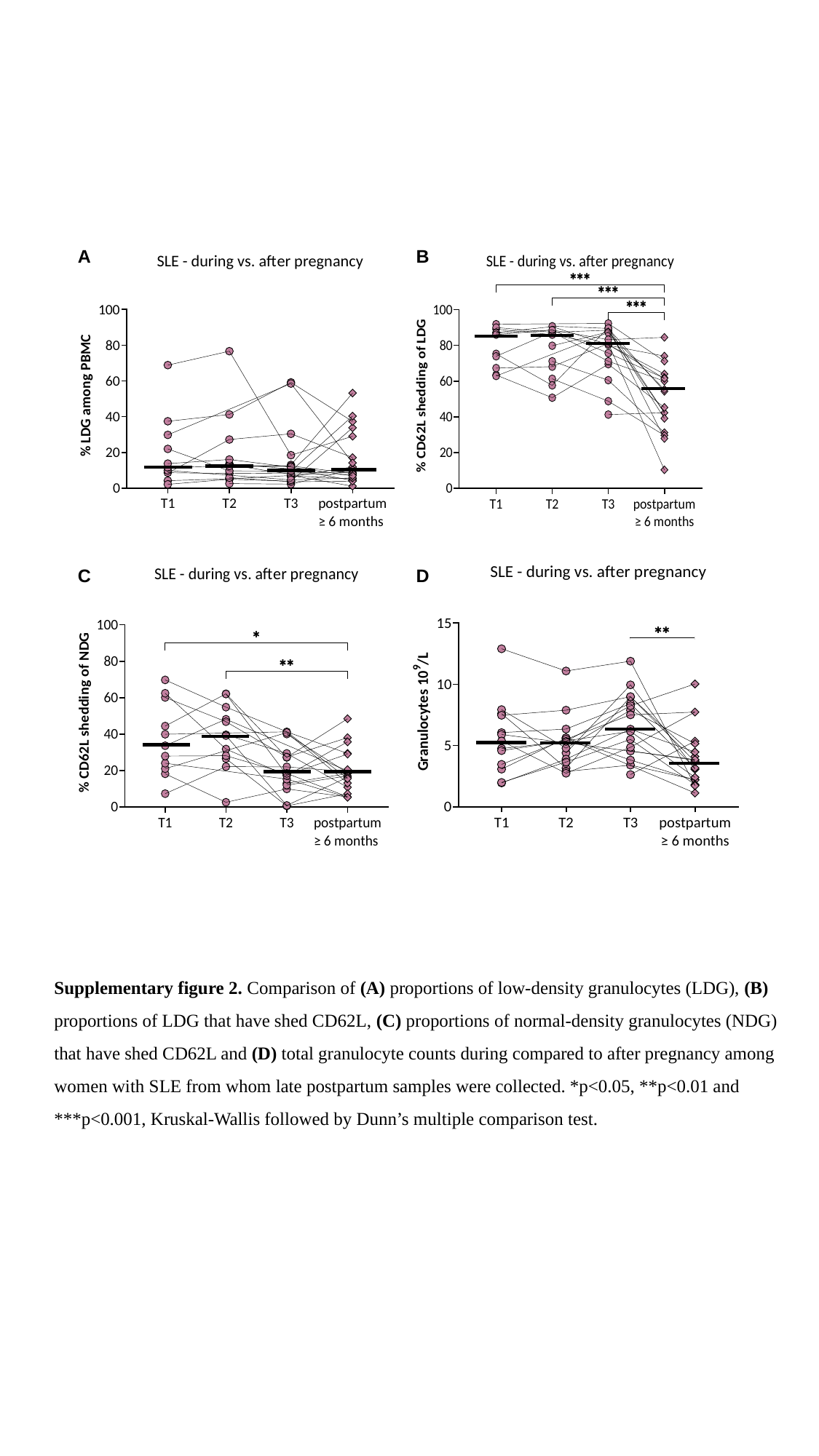

A
B
C
D
Supplementary figure 2. Comparison of (A) proportions of low-density granulocytes (LDG), (B) proportions of LDG that have shed CD62L, (C) proportions of normal-density granulocytes (NDG) that have shed CD62L and (D) total granulocyte counts during compared to after pregnancy among women with SLE from whom late postpartum samples were collected. *p<0.05, **p<0.01 and ***p<0.001, Kruskal-Wallis followed by Dunn’s multiple comparison test.

## Slide 4
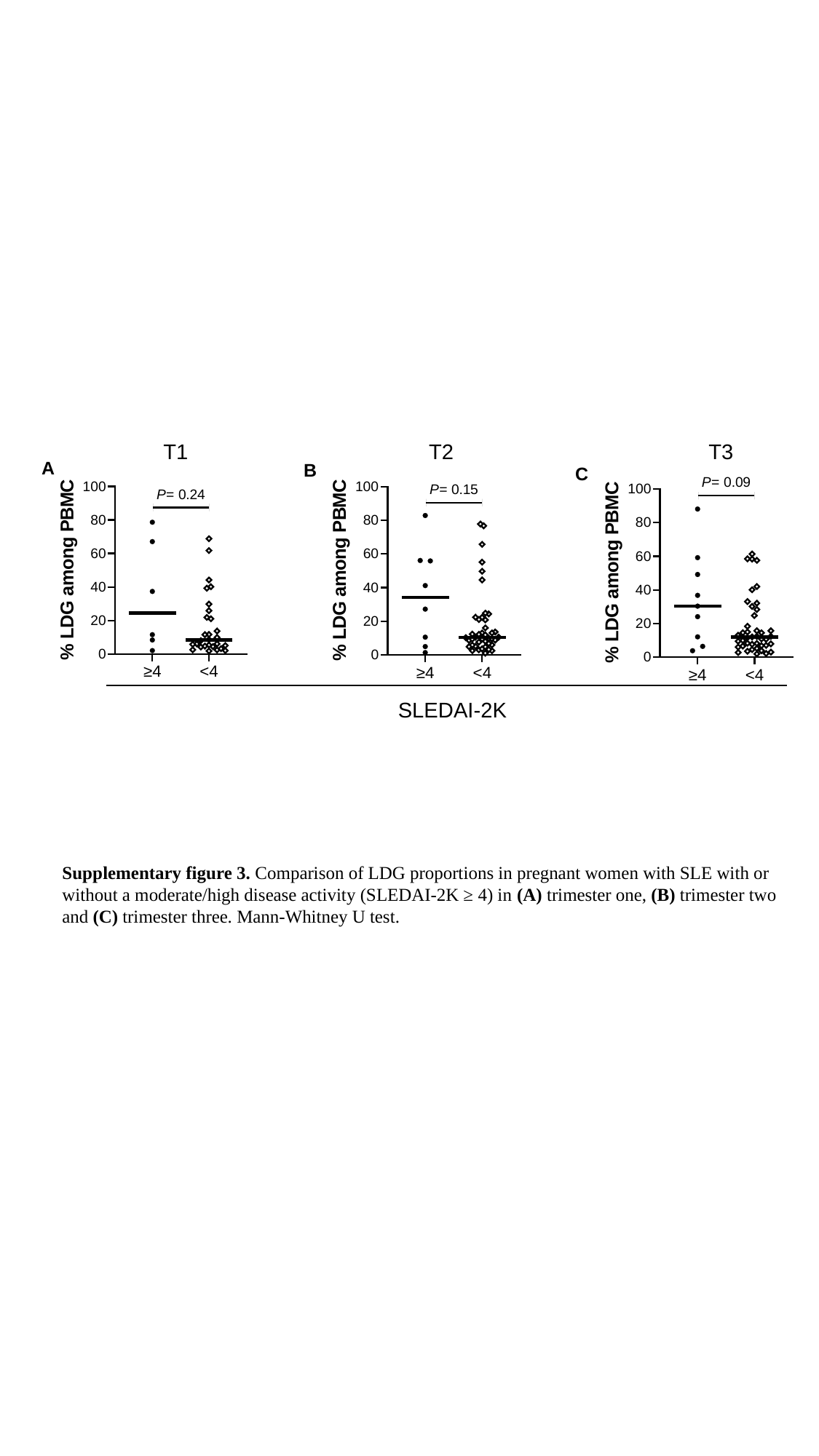

T1
T2
T3
A
B
C
SLEDAI-2K
Supplementary figure 3. Comparison of LDG proportions in pregnant women with SLE with or without a moderate/high disease activity (SLEDAI-2K ≥ 4) in (A) trimester one, (B) trimester two and (C) trimester three. Mann-Whitney U test.

## Slide 5
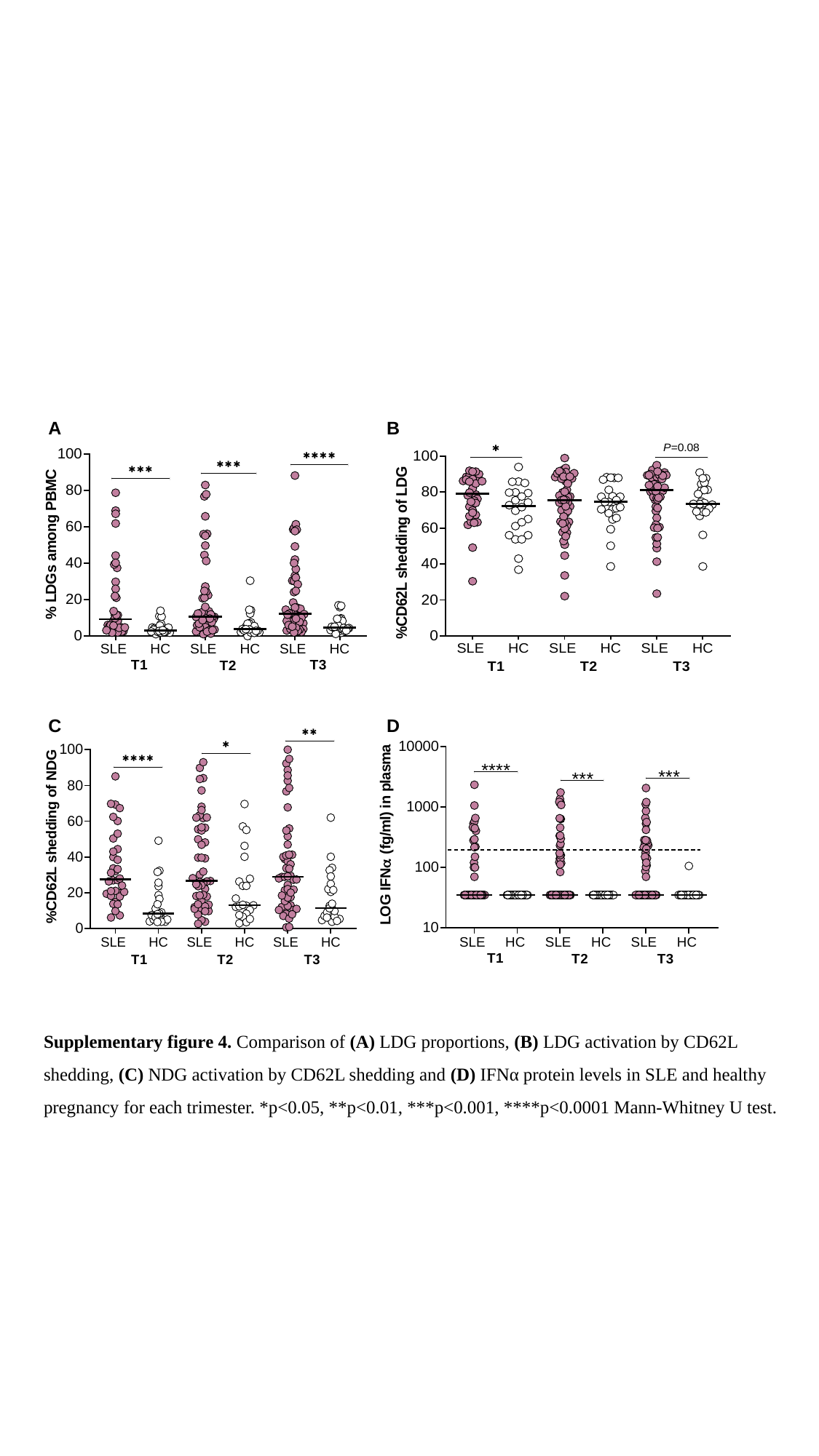

A
B
C
D
Supplementary figure 4. Comparison of (A) LDG proportions, (B) LDG activation by CD62L shedding, (C) NDG activation by CD62L shedding and (D) IFNα protein levels in SLE and healthy pregnancy for each trimester. *p<0.05, **p<0.01, ***p<0.001, ****p<0.0001 Mann-Whitney U test.

## Slide 6
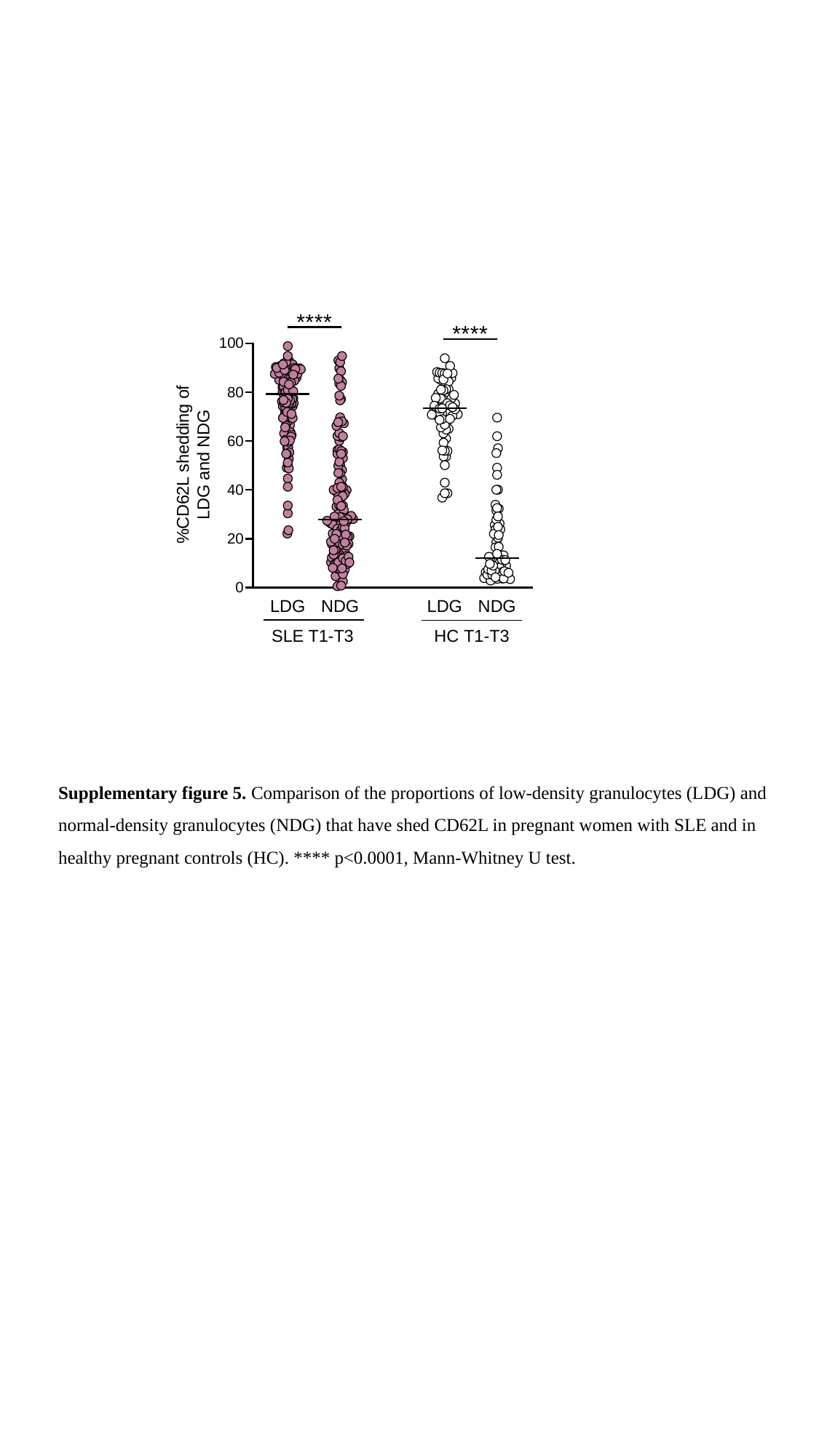

Supplementary figure 5. Comparison of the proportions of low-density granulocytes (LDG) and normal-density granulocytes (NDG) that have shed CD62L in pregnant women with SLE and in healthy pregnant controls (HC). **** p<0.0001, Mann-Whitney U test.

## Slide 7
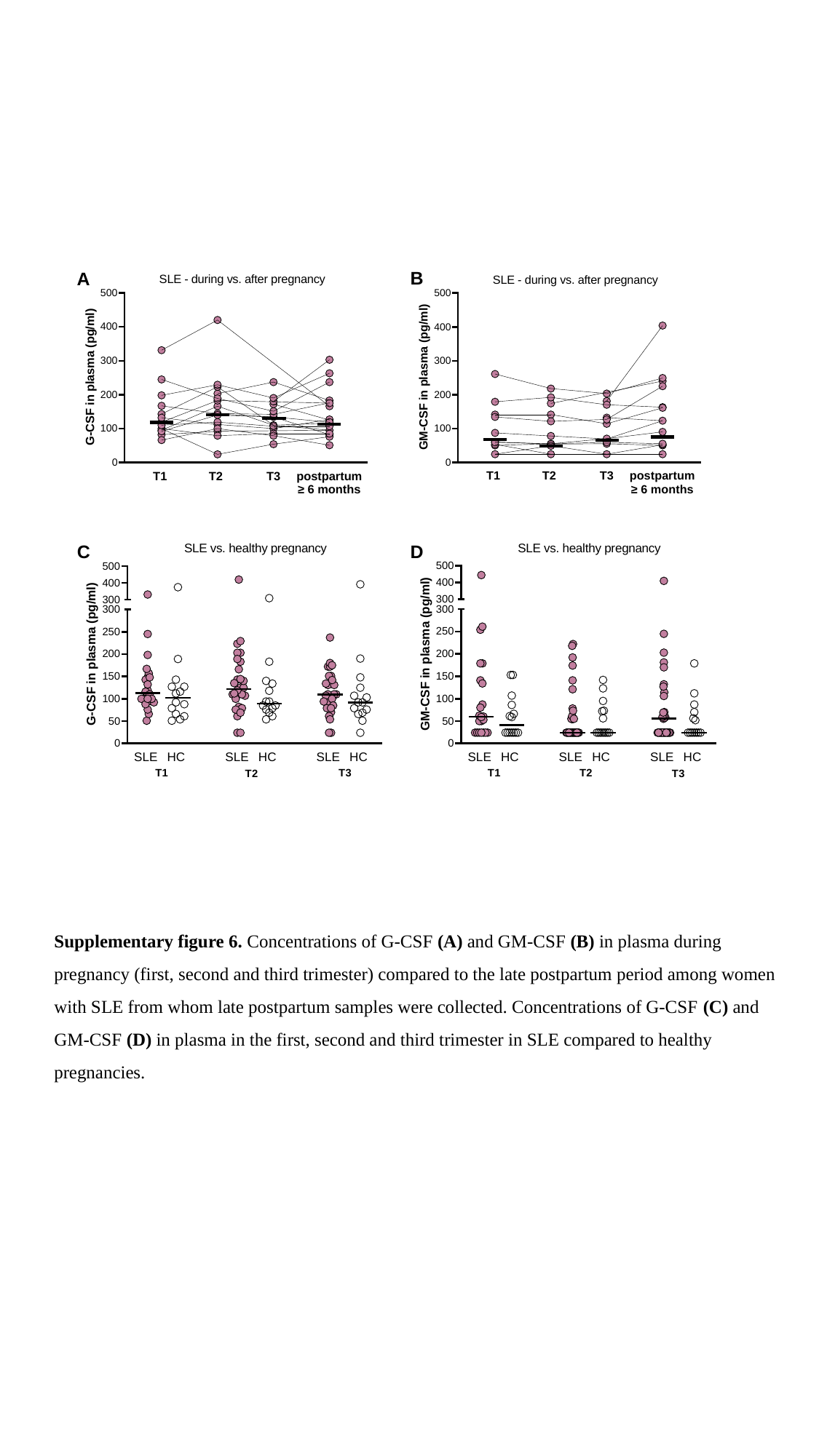

B
A
C
D
Supplementary figure 6. Concentrations of G-CSF (A) and GM-CSF (B) in plasma during pregnancy (first, second and third trimester) compared to the late postpartum period among women with SLE from whom late postpartum samples were collected. Concentrations of G-CSF (C) and GM-CSF (D) in plasma in the first, second and third trimester in SLE compared to healthy pregnancies.

## Slide 8
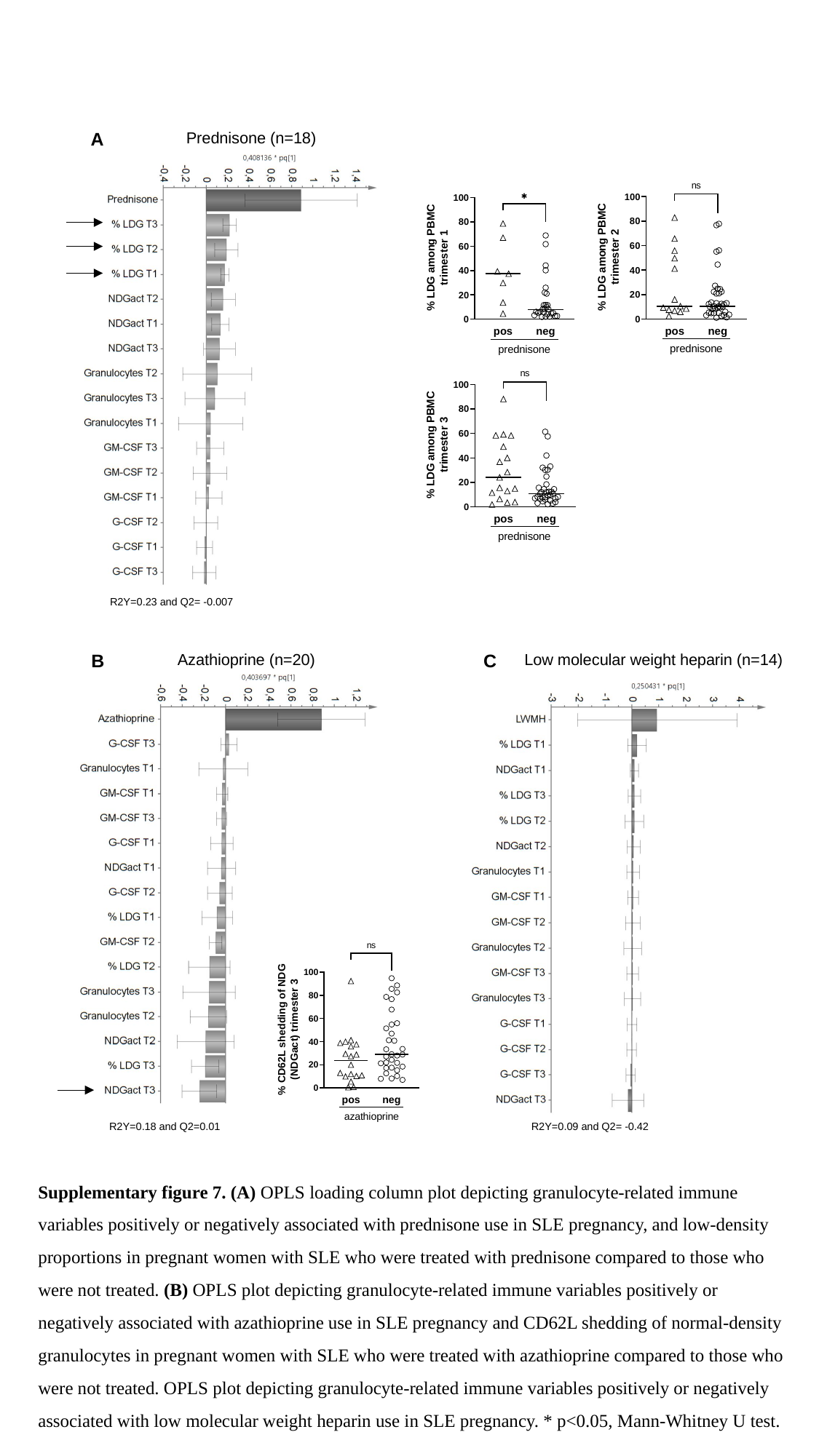

A
Prednisone (n=18)
R2Y=0.23 and Q2= -0.007
C
B
Azathioprine (n=20)
Low molecular weight heparin (n=14)
R2Y=0.18 and Q2=0.01
R2Y=0.09 and Q2= -0.42
Supplementary figure 7. (A) OPLS loading column plot depicting granulocyte-related immune variables positively or negatively associated with prednisone use in SLE pregnancy, and low-density proportions in pregnant women with SLE who were treated with prednisone compared to those who were not treated. (B) OPLS plot depicting granulocyte-related immune variables positively or negatively associated with azathioprine use in SLE pregnancy and CD62L shedding of normal-density granulocytes in pregnant women with SLE who were treated with azathioprine compared to those who were not treated. OPLS plot depicting granulocyte-related immune variables positively or negatively associated with low molecular weight heparin use in SLE pregnancy. * p<0.05, Mann-Whitney U test.

## Slide 9
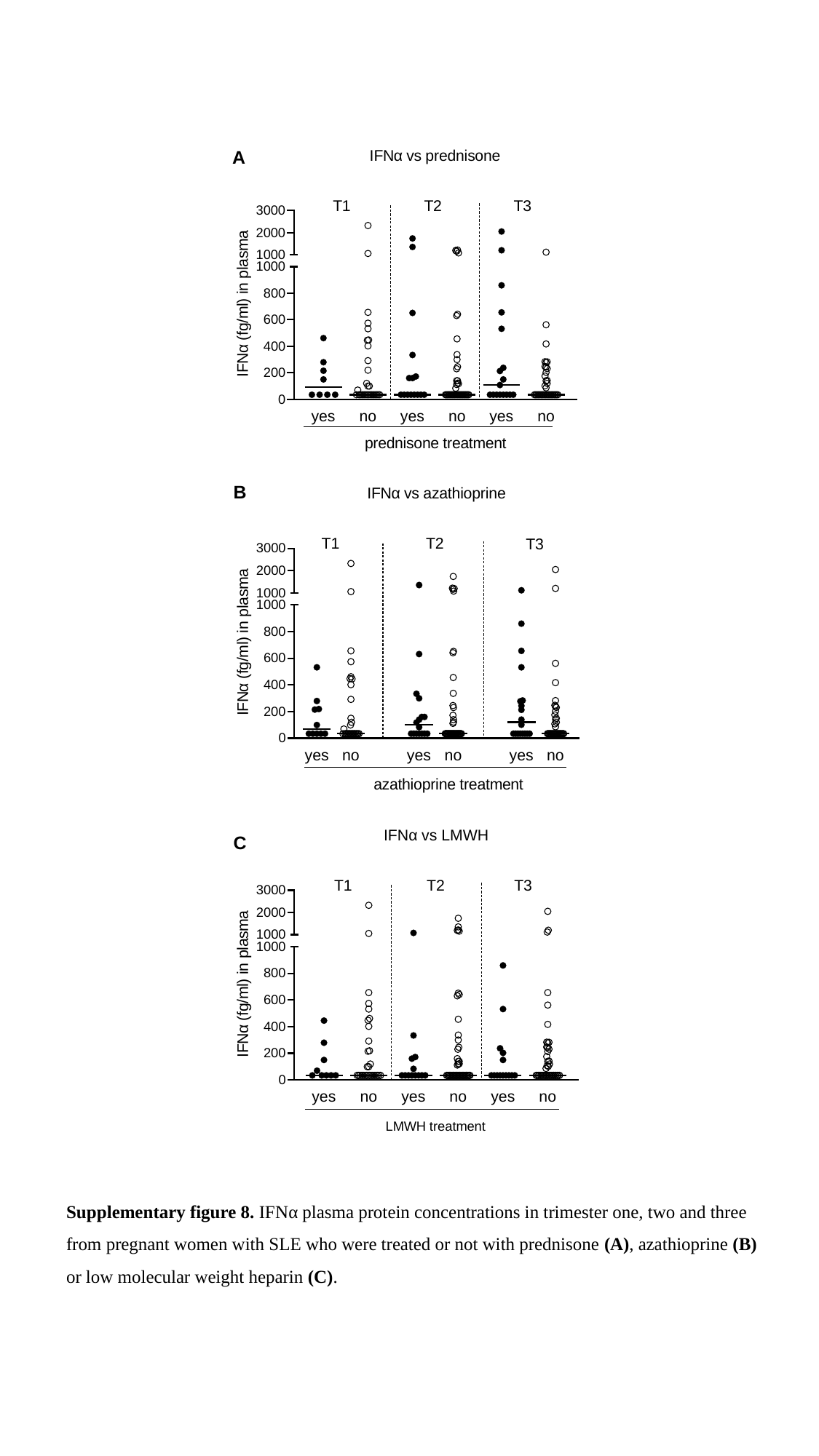

A
B
C
Supplementary figure 8. IFNα plasma protein concentrations in trimester one, two and three from pregnant women with SLE who were treated or not with prednisone (A), azathioprine (B) or low molecular weight heparin (C).

## Slide 10
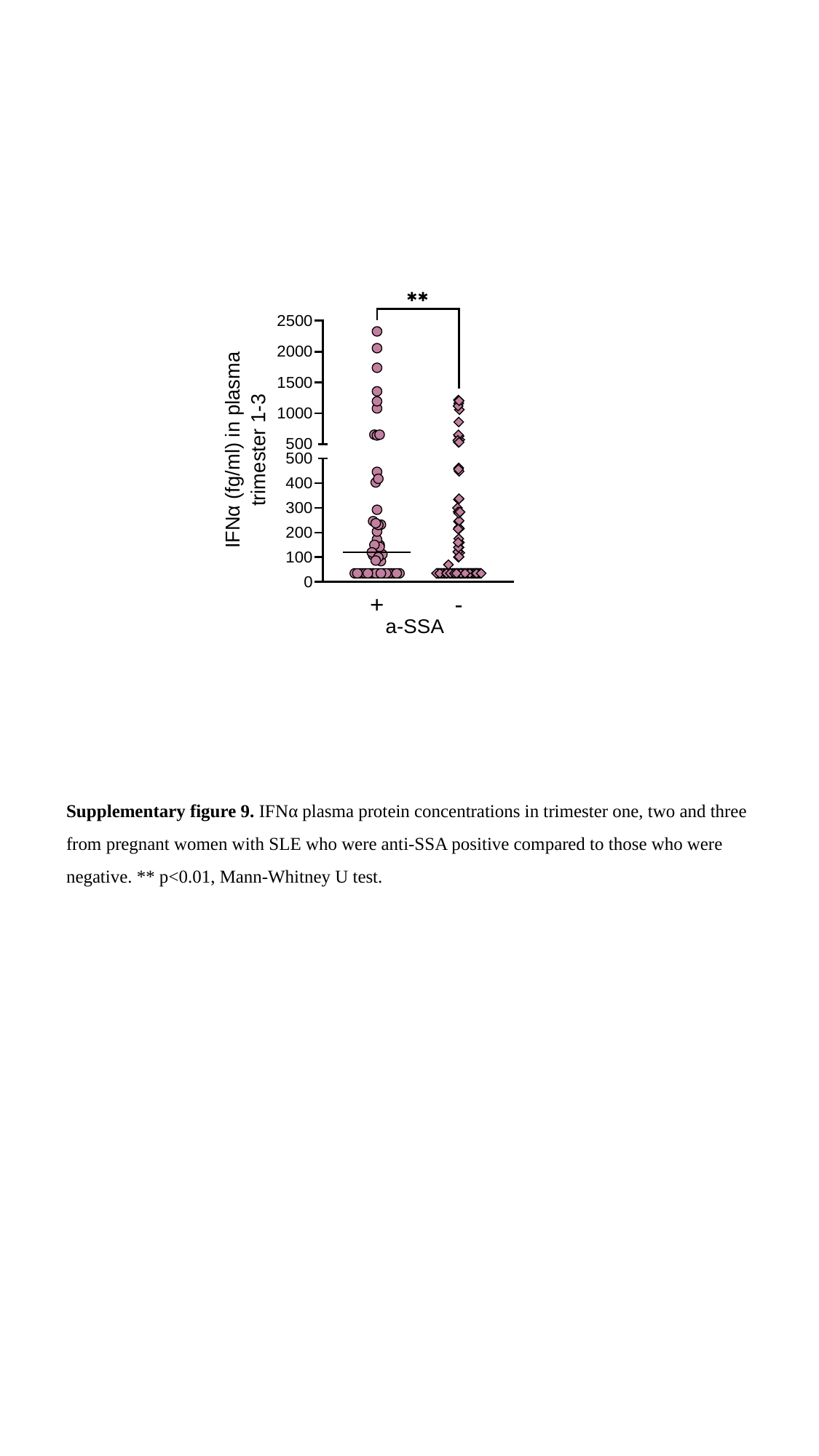

Supplementary figure 9. IFNα plasma protein concentrations in trimester one, two and three from pregnant women with SLE who were anti-SSA positive compared to those who were negative. ** p<0.01, Mann-Whitney U test.

## Slide 11
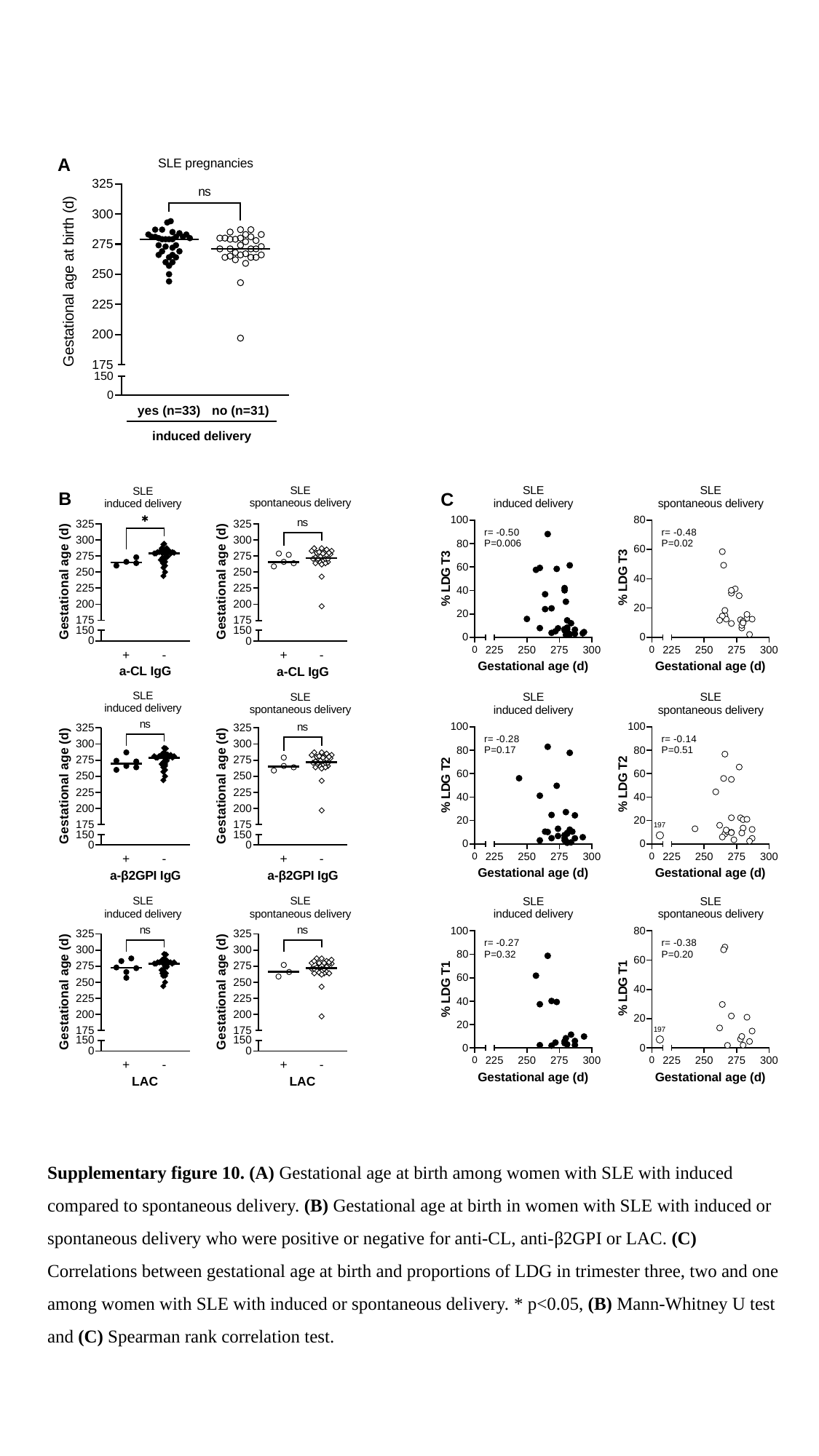

A
B
C
Supplementary figure 10. (A) Gestational age at birth among women with SLE with induced compared to spontaneous delivery. (B) Gestational age at birth in women with SLE with induced or spontaneous delivery who were positive or negative for anti-CL, anti-β2GPI or LAC. (C) Correlations between gestational age at birth and proportions of LDG in trimester three, two and one among women with SLE with induced or spontaneous delivery. * p<0.05, (B) Mann-Whitney U test and (C) Spearman rank correlation test.
