# Supplementary material for: Low-density granulocytes are related to shorter pregnancy duration but not to interferon alpha protein blood levels in systemic lupus erythematosus
Source: Arthritis Res Ther. 2023 Jun 22;25:107. doi: 10.1186/s13075-023-03092-w (PMC10286457; doi:10.1186/s13075-023-03092-w)
Supplement: Supplementary file 2 — Additional file 2: Supplementary Table 1. Numbers of collected blood samples. Supplementary Table 2. Antibodies used for flow cytometry. [file 13075_2023_3092_MOESM2_ESM.docx]

## **ADITIONAL FILE 2**

**Contains supplementary data to the following manuscript:**

**Low-density granulocytes are related to shorter pregnancy duration but not to interferon alpha protein blood levels in systemic lupus erythematosus**

^1*^Agnes Torell ^1,2^Marit Stockfelt ^1^Gunilla Larsson ^3^Kaj Blennow ^3,4,5,6,7,8^Henrik Zetterberg ^9^Dag Leonard ^9^Lars Rönnblom ^10^Muna Saleh ^10^Christopher Sjöwall ^11^Helena Strevens ^12^Andreas Jönsen ^12^Anders A. Bengtsson ^2^Estelle Trysberg ^13^Maria Majcuk Sennström ^14^Agneta Zickert ^14^Elisabet Svenungsson ^14^Iva Gunnarsson ^15^Karin Christenson ^15^Johan Bylund ^16,17,18^Bo Jacobsson ^1^Anna Rudin ^1^Anna-Carin Lundell

^1^Department of Rheumatology and Inflammation Research, Institute of Medicine, Sahlgrenska Academy at the University of Gothenburg, Sweden; ^2^Rheumatology, Sahlgrenska University Hospital, Gothenburg, Sweden; ^3^Department of Psychiatry and Neurochemistry, Institute of Neuroscience and Physiology, Sahlgrenska Academy at the University of Gothenburg, Mölndal, Sweden; ^4^Clinical Neurochemistry Laboratory, Sahlgrenska University Hospital, Mölndal, Sweden; ^5^Department of Neurodegenerative Disease, UCL Institute of Neurology, Queen Square, London, United Kingdom; ^6^UK Dementia Research Institute at UCL, London, United Kingdom; ^7^Hong Kong Center for Neurodegenerative Diseases, Clear Water Bay, Hong Kong, China; ^8^Winsconsin Alzheimer’s Disease Research Center, University of Wisconsin School of Medicine and Public Health, University of Wisconsin-Madison, Madison, WI, USA; ^9^Department of Medical Sciences, Rheumatology, Uppsala University, Uppsala, Sweden; ^10^Division of Inflammation and Infection, Department of Biomedical and Clinical Sciences, Linköping University, Linköping, Sweden; ^11^Department of Obstetrics and Gynecology, Institute of Clinical Sciences, Skåne University Hospital, Lund, Sweden; ^12^Department of Clinical Sciences Lund, Rheumatology, Lund University, Skåne University Hospital, Lund, Sweden; ^13^Department of Womens and Childrens Health, Division for Obstetrics and Gynecology, Karolinska University Hospital, Karolinska Institute, Stockholm, Sweden; ^14^Department of Medicine Solna, Division of Rheumatology, Karolinska Institute, Karolinska University Hospital, Stockholm, Sweden; ^15^Department of Oral Microbiology and Immunology, Institute of Odontology, Sahlgrenska Academy at the University of Gothenburg, Sweden; ^16^Department of Obstetrics and Gynecology, Institute of Clinical Sciences, Sahlgrenska Academy at the University of Gothenburg, Gothenburg Sweden; ^17^Department of Obstetrics and Gynecology, Sahlgrenska University Hospital, Gothenburg Sweden; ^18^Department of Genetics and Bioinformatics, Domain of Health Data and Digitalisation, Institute of Public Health, Oslo, Norway.

**Running title:** Low-density granulocytes in SLE pregnancy

## **Supplementary Table 1.** Numbers of collected blood samples

|  | Trimester one  week 10-12 | Trimester two  week 18-20 | Trimester three  week 32-34 | Late postpartum  ≥ 6 months after delivery |
| --- | --- | --- | --- | --- |
| SLE (n=69) | 39 | 57 | 60 | 19 |
| HC (n=27) | 22 | 26 | 24 |  |

## **Supplementary Table 2.** Antibodies used for flow cytometry

| Flow cytometry reactivity | Assay | Conjugate | Clone | Company |
| --- | --- | --- | --- | --- |
| CD62L | LDG/NDG | PE | SK11 | BD Biosciences |
| CD45 | LDG/NDG | FITC | 2D1 | Biolegend |
| CD15 | LDG/NDG | PE-Cy7 | HI98 | BD Biosciences |
| CD14 | LDG/NDG | APC | M5E2 | BD Biosciences |
| CD10 | LDG/NDG | BV510 | HI10a | BD Biosciences |
| CD45 | TruCount | PerCP | 2D1 | BD Biosciences |
